# Optimization strategies for elderly hearing aid user satisfaction based on the Kano QFD integrated model and FAD theory

**DOI:** 10.1038/s41598-025-17546-5

**Published:** 2025-10-02

**Authors:** Yansheng Ren, Kangheui Cha

**Affiliations:** Industrial Design Department, Hongik University, Seoul, 04066 Korea

**Keywords:** Kano QFD, Triangular fuzzy numbers, Axiomatic design, Senior hearing aids, User satisfaction, Mechanical engineering, Electrical and electronic engineering

## Abstract

Advancements in global healthcare and declining birth rates have accelerated population aging, intensifying challenges such as workforce shortages and increasing the demand for tailored solutions to enhance the quality of life for the elderly. This study addresses a critical gap in the design of smart products for seniors by proposing a two-stage user satisfaction framework that integrates the Kano model, Quality Function Deployment (QFD), and Fuzzy Axiomatic Design (FAD) to manage uncertainties in user evaluations. Using a case study of Senior Hearing Aids (SHAs), the framework identifies nine key design factors and prioritizes improvements based on Importance Coefficients (ICs): environmental mode audio noise reduction (FR_7_, IC = 0.9140), battery systems (FR₄, IC = 0.3416) for extended usage, and lightweight structure (FR₃, IC = 0.3219) for comfort. Secondary improvements focus on modular design (FR₅) and audio components (FR₆) to ease maintenance and enhance sound clarity. Lower-priority features, such as simple button interaction and easy-to-remove components, are optimized through integrated design strategies. Uncertainty analysis using FAD, combined with expert validation, demonstrates the framework’s practicality and offers a scalable approach for aging-focused product design, with broader implications for smart healthcare innovations.

**Supplementary Information:**

The online version contains supplementary material available at 10.1038/s41598-025-17546-5.

## Introduction

With the rapid growth of the global elderly population, the proportion of individuals aged ≥ 65 is projected to increase from 10% in 2022 to 16% by 2050^1^. Health issues, like hearing loss, become increasingly prevalent with aging. Consequently, there has been an increase in the demand for smart wearable hearing aids, characterized by their convenience and intelligent features, making them a popular choice for product design. The use of smart wearable devices can address the issues associated with age-related hearing impairments in the daily lives of elderly individuals, thereby improving their quality of life. At present, hearing aids are the most economical and effective solution for addressing age-related hearing loss and maintaining normal social activities. However, the enclosed design of hearing aid molds can amplify low-frequency self-generated sounds, such as speaking, chewing, and swallowing. Moreover, the occlusive earplugs can make the user’s voice sound unnatural (muffled and hollow)^2^. Additionally, non-users often cite external factors as reasons for not using hearing aids, while family members may refrain from using them due to attitudinal barriers. Both hearing aid users and their family members state device-related factors as obstacles to usage^3^. Therefore, our goal was to design senior hearing aids (SHAs) for elderly users to mitigate these problems.

The SHA designs must consider both quantitative (e.g., weight and cost) and qualitative (e.g., anti-slip properties, comfort, and ease of operation) criteria. Qualitative criteria are complex, interdependent, and often vaguely defined, making hearing aid evaluation a fuzzy multi-criteria decision-making (MCDM) problem. Axiomatic design (AD) is a prescriptive engineering design theory that provides a systematic foundation for design decisions^4^which is used in the context of MCDM. In AD, two axioms enable designers to formalize design problems and solutions at the conceptual stage to eliminate poor designs, select the best proposals, and improve existing designs^5^. Based on the complexity and uncertainty of practical user needs, Cebi et al.^6^ discussed the adaptability of existing AD principles to fuzzy set theory, and proposed a combined fuzzy axiomatic design (FAD) to address linguistic evaluations^7^. FAD simultaneously processes quantitative and qualitative data to rank alternatives based on given criteria. Kulak et al.^8^ analyzed various articles on AD methods published between 1990 and 2009, covering design types, application domains, methodologies, and evaluation types. Feng^9^ reviewed relevant FAD studies that employed crisp numbers, interval numbers, hesitant fuzzy sets, intuitionistic fuzzy sets, and linguistic variables as decision information. Table 1 summarizes FAD studies published between 2018 and 2023.

**Table 1:** Summary of FAD studies conducted between 2018 and 2023.

Authors	Fuzzy numbers	Integrated methods	Application type	Problem
Kahraman et al^65^	TrFN	N/A	Real case	Multicriteria landfill site selection
Maghsoodi et al^66^	TFN	FBWM	Real case	Selection of a conceptual prototype for a loudspeaker
Xingli Wu et al^67^	TFN	ORESTE	Real case	Application in supply chain finance decision-making with credit risk assessments
Desmond Eseoghene Ighravwe et al^68^	TrFN	AHP and WASPAS	Real case	Proactive maintenance strategy selection for manufacturing systems
Jianghong Feng^9^	TFN	AHP	Real case	Wind farm site selection from the perspective of sustainability
Mumtaz Karatas^62^	TFN	AHP	Illustrative case	Selection of hydrogen energy storage
Jin Ye et al^69^	TFN, TrFN, and IN	3WD	Illustrative case	Novel decision-making method
Ai-Hua Liu et al^70^	LFTDs	HMCGDM	Real case	Heterogeneous multi-criteria group decision-making with linguistic fuzzy truth degrees
Jianghong Feng^71^	TFN	LEW	Illustrative case	Site selection for electric-vehicle charging stations from a sustainable perspective
Gulumser et al^72^	TFN	AHP	Real case	Photocatalyst selection
Ilker Gölcük^73^	IT2F	BWM	Illustrative case	evaluating blockchain deployment projects in the supply chain
Qinghua Liu et al^74^	SFPR	AHP	Illustrative case	Human-machine interface design evaluation
Ismail Kandemir et al^75^	TFN	N/A	Illustrative case	Development of an instructional design model selection approach for maritime education and training

**Notes**: TrFN: trapezoidal fuzzy numbers; TFN: triangular fuzzy numbers; IN: interval numbers; LFTDs: linguistic fuzzy truth degrees; IT2F: interval type-2 fuzzy numbers; SFPR: simple fuzzy pattern recognition; AHP: analytic hierarchy process; WASPAS: weighted aggregated sum product assessment; 3WD: three-way decision; HMCGDM: hybrid multi-criteria group decision making; LEW: linguistic entropy weight; and BWM: best-worst method.

As seen in Table 1, most FAD studies employ triangular fuzzy numbers (TFNs) and trapezoidal fuzzy numbers (TrFNs) in combination with classical MCDM methods, such as analytic hierarchy process (AHP), three-way decision (3WD), multi-objective optimization by ratio analysis (MULTI-MOORA), grey relational analysis (GRA), and criteria importance through intercriteria correlation (CRITIC). These methods have been applied across various fields, including mechanical, electronic, chemical, design, engineering, and manufacturing industries.

Practical product design, irrespective of the industry, ultimately hinges on the effective satisfaction of user needs. Consequently, user satisfaction remains a core and critical factor in product development. User satisfaction refers to the overall user perception and evaluation of their experience with a product or service, reflecting whether it meets their needs and expectations. High user satisfaction typically indicates that the product or service meets/exceeds expectations, resulting in a pleasant user experience. Conversely, low user satisfaction suggests that the product or service does not meet user expectations, potentially leading to customer attrition. User satisfaction is multidimensional and varies with different product designs. Therefore, understanding and integrating these dimensions into the product design process is crucial for enhancing the overall user experience^10^. In general, when users evaluate product performance, they focus on the value offered by individual functions rather than the overall performance; however, the definition, measurement, and specifications of each function differ across products. Therefore, there is a need for a comprehensive and universally applicable method for evaluating these functions. Parasuraman et al.^11^ first introduced the concept of a user satisfaction index and developed the SERVQUAL scale, which measures the gap between customer expectations and actual perceptions through five dimensions: reliability, responsiveness, assurance, empathy, and tangibles. In 1994, the United States established the American Customer Satisfaction Index (ACSI)^12^. Zhan et al.^13^ constructed a structural model based on the Place Attachment theory and the ACSI to examine the relationships between exhibition attachment, customer satisfaction, complaints, and loyalty. Othman et al. utilized smart initiatives to develop a model for simple and continuous assessment of user satisfaction, exploring the ability of temporal parameters to monitor and build performance^14^. Li et al.^15^ developed a two-stage nonlinear user satisfaction decision model with a generalizable framework. This model, based on the Kano mapping rules, describes the post-purchase evaluation behavior over non-compensatory and compensatory stages. In the non-compensatory stage, initial user satisfaction is influenced by basic attributes, while in the compensatory stage, final user satisfaction is influenced by the collective performance of other attributes. Kwon et al.^16^ established hypothetical relationships between (i) latent factors and user satisfaction with battery electric vehicles (BEVs), and (ii) BEV usage satisfaction and repurchase/recommendation intentions. Onodera et al.^17^ proposed an innovative evaluation framework based on user-centric assessment methods to accurately determine user satisfaction. Liang et al.^18^ applied AHP, distributed linguistic representations, and probabilistic linguistic term sets to evaluate customer satisfaction with online-to-offline food delivery services.

Although existing studies have significantly advanced the measurement and modeling of user satisfaction dimensions, a critical gap remains in systematically integrating these insights into engineering design frameworks. Current satisfaction models—such as SERVQUAL, ACSI, and Kano-based approaches—are primarily employed during the evaluation phase of product design. However, they lack a robust mechanism for translating multidimensional satisfaction criteria into quantifiable design range during the early stages of development. This disconnect is particularly problematic in complex user-centered systems such as hearing aids, where physiological adaptation requirements (e.g., compensation for age-related hearing loss) and technical constraints (e.g., miniaturization) demand precise mapping between satisfaction attributes and axiomatic design decisions.

This theoretical-practical divide is especially evident in the design research of SHAs. Although some methods attempt to bridge the gap between user needs and engineering specifications, their effectiveness is limited by the absence of mechanisms to capture dynamic user requirements and by obstacles to interdisciplinary collaboration. These limitations prevent the full alignment of technical solutions with the evolving needs of aging populations.

Current SHA design research generally falls into two major categories, one of which is clinical audiology-based design. However, this design pathway presents significant limitations. Current SHA design approaches still fall short in addressing the diverse needs of elderly users. On one hand, most existing studies rely on static user research methods, neglecting the evolving needs that occur over prolonged use. For instance, Ng et al. noted that user satisfaction with hearing aids often differs markedly between the initial period of use and several months later, revealing a trend of dynamically changing needs^19^. Moreover, Takaki et al. further emphasized that user experience is a dynamically evolving process, requiring continuous product updates at different usage stages to maintain satisfaction^20^. On the other hand, the hearing aid experience is not solely influenced by auditory functions but also involves multisensory integration factors such as visual cues, tactile feedback, and cognitive load. However, most current design approaches focus primarily on optimizing electroacoustic performance and lack interdisciplinary collaboration, making it difficult to comprehensively enhance the user experience^21^. In their study on modular hearing aid design, Heiss et al. pointed out that the absence of effective communication mechanisms across disciplines in the current development process limits the potential for innovation in user experience^22^. Additionally, Dritsakis et al. emphasized that promoting interdisciplinary collaboration in audiological research can significantly improve the feasibility of interventions and overall user satisfaction^23^. Furthermore, Young et al. argued that the introduction of cross-disciplinary approaches such as design thinking helps better identify genuine user needs and enables proactive optimization at the early stages of product development^24^. These issues reveal two key research gaps: (1) the absence of mechanisms for dynamic demand mapping, which causes design iterations to lag behind users’ physiological changes; and (2) the lack of interdisciplinary collaboration frameworks, leading to a disconnect between technical development and the specific demands of gerontological engineering.

Despite its advantages, FAD faces several limitations that reduce its effectiveness in user-centric design:

1. Inadequate Identification of Customer Needs: FAD emphasizes technical parameters and design characteristics but often overlooks comprehensive early-stage analysis of customer requirements, resulting in inaccurate translation into actionable design inputs.
2. Barriers to Cross-Departmental Collaboration: Information distortion during interdepartmental communication creates asymmetry in understanding, undermining consistency in decision-making and complicating the alignment between technical and user needs.
3. Gaps in Feature Prioritization: FAD does not provide a comprehensive mechanism for prioritizing design features, which may lead to misaligned resource allocation and compromise the delivery of user-centered value.

To address these challenges, future research should focus on strategies to refine user needs into quantifiable design range or incorporate MCDM methods—such as Quality Function Deployment (QFD)—to improve cross-functional collaboration and ensure that design features align closely with user expectations. This study proposes a two-stage user satisfaction framework that integrates QFD and FAD to overcome the above limitations. The first stage utilizes a tailored QFD model for SHAs to systematically analyze user needs and facilitate interdepartmental collaboration, thereby optimizing the design process and enhancing both product quality and user satisfaction. In the second stage, the FAD model identifies gaps between product attributes and user satisfaction based on performance standards set by the company, highlighting areas that require critical improvement and helping avoid excessive investment in low-priority features.

The paper is structured as follows:

Section “Introduction”: Introduces the research methodology, reviews relevant literature, and outlines the foundational concepts of Axiomatic Design (AD), FAD, and QFD;

Section "Research Background And Methodology": Describes the development of the two-stage QFD–FAD framework, with emphasis on its application to SHA design;

Section "Two-stage framework design": Validates the framework’s applicability and effectiveness through a case study, using comparative analysis and sensitivity assessments;

Section “Case study”: Discusses key findings and managerial implications for aging-oriented product design;

Section "Data validation and discussion": Concludes the study, outlines its limitations, and proposes directions for future research, particularly concerning the enhancement of FAD integration with user-centric methodologies.

## Research background and methodology

This study involved human participants and adhered to all applicable ethical guidelines and regulations. Ethical approval was obtained from the School of Industrial Design at Hongik University. All procedures were conducted in accordance with relevant ethical standards, including the principles outlined in the Declaration of Helsinki. Informed consent was obtained from all participants prior to their involvement. Participants were fully informed of the study’s purpose, procedures, and their rights, ensuring voluntary participation without any form of coercion.

This study employed four key methodological tools: the Kano Model, QFD, FAD, and TFN. The Kano Model is a tool for evaluating and classifying customer needs, helping to identify product or service features that significantly enhance user satisfaction. QFD is a structured approach for translating customer demands into technical specifications, ensuring that the voice of the customer is fully addressed in the design process. FAD is a system design framework based on fuzzy logic principles, well-suited for handling uncertainty in decision-making. TFN represents uncertain or vague data using triangular fuzzy numbers, assisting in the evaluation of user needs and preferences to more effectively guide the product design process.

### Evaluation criteria for SHAs

**Fig. 1:**
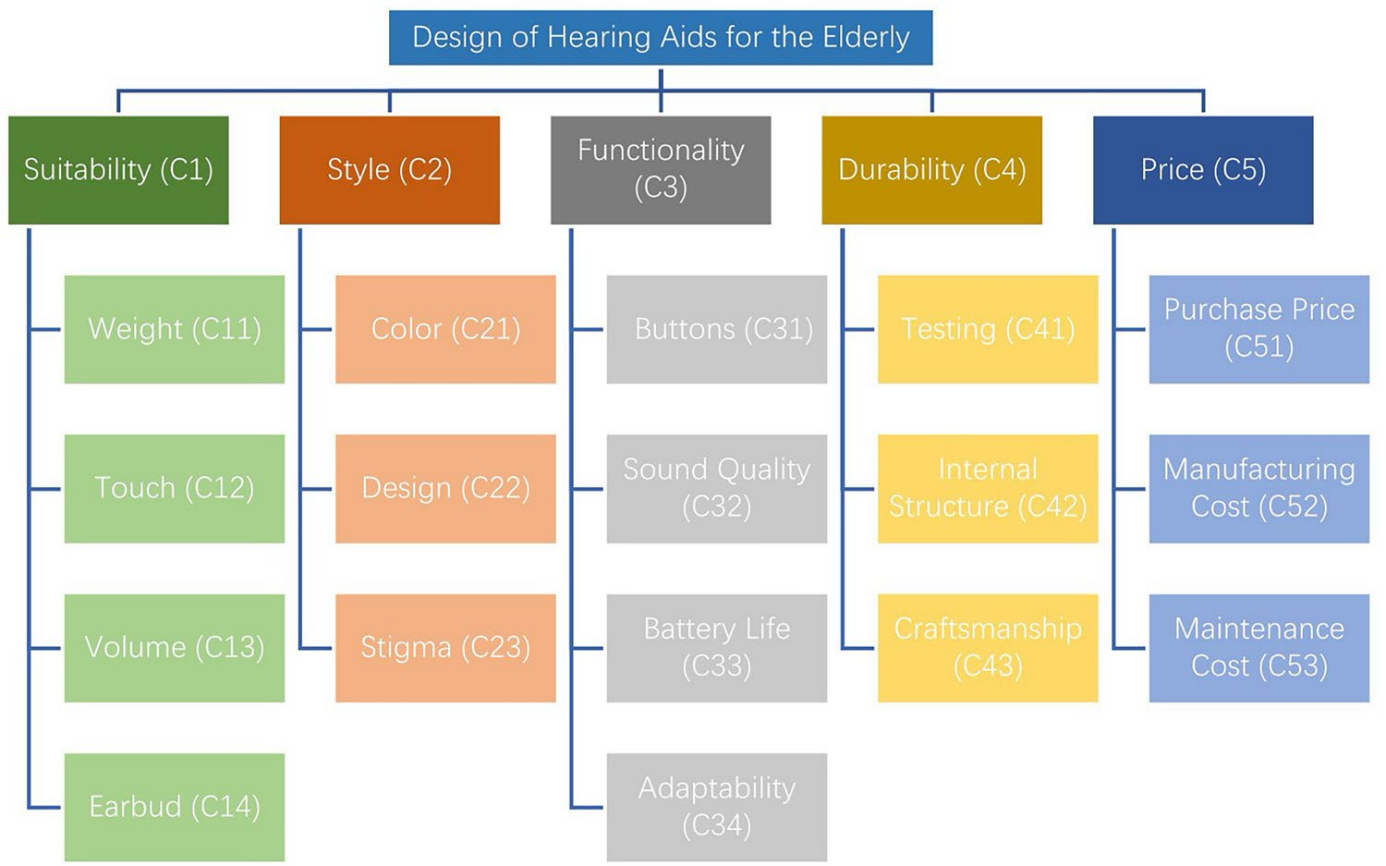
Evaluation criteria for SHA design.

SHAs enable elderly users to lead active lives. Numerous studies have proposed evaluation criteria for hearing aids, including Chi-Hung Lo, Larissa Bannon et al., Alinka Greasley et al., Chengmin Zhou et al., Ken Takaki et al., Yin Liu et al., Elizabeth Convery et al., Sergei Kochkin et al., Zhengjun Zhou et al., Majid H. Alsulami et al., Rebecca J. Bennett et al., Elke M. J. Devocht et al., Erik J. Jorgensen et al., Robyn M. Cox et al., Solveig C. Voss et al., Jens Cubick et al., Erin M. Picou et al., Stuart Gatehouse, Lucas Kwai Hong Lui et al., and Yan Yan et al.^20,25–43^. The MarkeTrak surveys offer consumer perspectives, elucidating factors associated with satisfaction^44^. Picou et al.^40^ identified six key factors influencing user satisfaction: performance, sound quality, professional support, physical attributes, ease of maintenance, and cost. Similarly, Kasemsiri et al.^45^ highlighted the degree of hearing loss, individual challenges, user motivation and expectations, personality, counseling, and economic considerations as factors affecting hearing aid selection. Although the aforementioned studies do not exclusively focus on SHAs, insights from users across a wide age range provide theoretical support for designing products tailored to older adults. Based on these studies, we identified 5 primary criteria for evaluating SHAs design for the elderly, namely suitability, style, functionality, durability, and price along with 17 sub-criteria (Fig. 1; Table 2).

**Table 2:** SHA evaluation criteria definitions.

Primary criteria	Sub-criteria	Definition	References
Suitability (C1)	Weight (C11)	Appropriateness of weight to reduce usage burden.	^45^
	Touch (C12)	Tactile comfort during use.	^45^
	Volume (C13)	Compactness and portability of design for ease of carrying and use.	^20^
	Earbud (C14)	Comfortability of earbud design based on elderly users’ ear shapes and skin sensitivity	^39^^43^,
Style (C2)	Color (C21)	Aesthetic color options catering to elderly preferences	^28^^32^,
	Design (C22)	Alignment with user preferences and product trends	^25^^29,39^,
	Stigma (C23)	Stigma related to wearing hearing aids and psychological acceptance	^25^
Functionality (C3)	Buttons (C31)	Logicality of button arrangement and ease of operation	^33^
	Sound quality (C32)	Clarity and quality of sound	^2,34,35,39,40,43,47,48,54^
	Battery life (C33)	Number of hours the battery lasts with complete charge	^26^^31^,
	Adaptability (C34)	Sound comfort and environmental adjustment	^26^
Durability (C4)	Testing (C41)	Compliance with safety and quality standards	^40^
	Internal structure (C42)	Internal component quality and reliability	^42^^43^,
	Craftsmanship (C43)	Material selection and production quality	^2^^42^,
Price (C5)	Purchase price (C51)	Competitiveness and reasonability of the cost	^31^
	Manufacturing Cost (C52)	Cost of manufacturing and assembly	^45^
	Maintenance cost (C53)	Affordability of repair and upkeep	^31^^37^,

### Related research

In product development, the Kano model and Quality Function Deployment (QFD) method are widely applied, and their combined use has been proven to be significantly effective in identifying and optimizing product design features. Numerous innovative cases based on these two models have emerged. For example, Wang et al. integrated Grounded Theory (GT) with the AHP-QFD method to successfully identify the key requirements for greenhouse pear pollination drones and proposed theoretical solutions, providing guidance for the intelligent development of agricultural machinery^46^. Additionally, they employed the KANO-AHP-FCE model to systematically evaluate the design requirements of walkers, helping to clarify key design elements and identify areas for improvement^47^. These theoretical studies have effectively enhanced the alignment between product design and user needs, providing strong theoretical support for improving the mobility safety of the elderly. Through these cases, we can see the significant theoretical value of user-centered design thinking in engineering practice, and they also provide theoretical guidance for the innovation of combining Kano and QFD.

Building on these theoretical studies, our proposed Kano–QFD–FAD integrated framework further expands this approach, providing a broader methodological context for applying user-centered design principles. To situate our proposed framework within a broader methodological context, we reviewed a range of interdisciplinary studies combining user-centered theory with engineering design methods. In recent years, the integration of the Kano Model and QFD has been increasingly applied in the field of smart products for the elderly. For instance, Li et al. optimized the user experience of smart refrigerators using the Kano–QFD approach, effectively improving elderly users’ satisfaction with smart home appliances^48^. Similarly, Cao et al. combined Kano with AHP and QFD to construct an interaction design system for elderly-friendly smartwatches^49^. These studies demonstrate that by identifying satisfaction drivers among elderly users, researchers have developed mechanisms for translating user needs into technical features, thereby optimizing both user experience and system usability.

Nevertheless, an integrated framework incorporating FAD into the Kano–QFD process is still lacking. FAD, grounded in the independence and information axioms, emphasizes the optimal configuration of design range under conditions of uncertainty. It is particularly suitable for perceptual and interaction-based products for the elderly, whose needs are often vague, dynamic, and difficult to quantify. Therefore, the Kano–QFD–FAD model proposed in this study extends the existing logic of emotional need classification and technical mapping by introducing FAD to provide both theoretical support and a computational pathway for system design in complex and ambiguous environments.

#### Kano model

The Kano model was developed by Noriaki Kano in 1984 to assess customer satisfaction and inform product development, classifying and prioritizing user needs into six categories: (i) Attractive attributes (A); (ii) one-dimensional attributes (O); (iii) must-be attributes (M); (iv) indifferent attributes (I); (v) reverse attributes (R); and (vi) questionable attributes (Q)^50^ (see Table 3). The Kano model was originally used to evaluate customer satisfaction. In this study, it is applied to categorize user needs and determine their relative importance. The model serves as a practical tool to identify which SHA features have the greatest impact on the satisfaction of elderly users.

**Table 3:** Kano evaluation criteria.

User Requirements		Attitude Toward: Reverse Problem				
		Satisfaction	Should-be	Indifferent	Tolerable	Dissatisfied
Attitude Toward: Positive Problem	Satisfaction	*Q*	*A*	*A*	*A*	*O*
	Should-be	*R*	*I*	*I*	*I*	*M*
	Indifferent	*R*	*I*	*I*	*I*	*M*
	Tolerable	*R*	*I*	*I*	*I*	*M*
	Dissatisfied	*R*	*R*	*R*	*R*	*Q*

By categorizing user needs, the Kano model clarifies which functions of SHA have a critical impact on customer satisfaction. These classification results provide a foundation for prioritization in the subsequent QFD process, enabling QFD to more effectively translate key user needs for SHA into specific engineering requirements. This ensures that design decisions for SHA align more closely with user expectations.

#### QFD

QFD is a common user-oriented quality management tool for product design and development. QFD translates customer needs and quality requirements into engineering characteristics (ECs). Originally a popular tool utilized in manufacturing processes^51^ QFD assists product design teams in both understanding customer requirements and market trends and effectively shortening product development timelines^52^. QFD systematically connects user needs with technical requirements, enabling designers to gain deeper insights into customer expectations and quickly respond to market dynamics to improve product quality across various industries^53–55^.

**Fig. 2:**
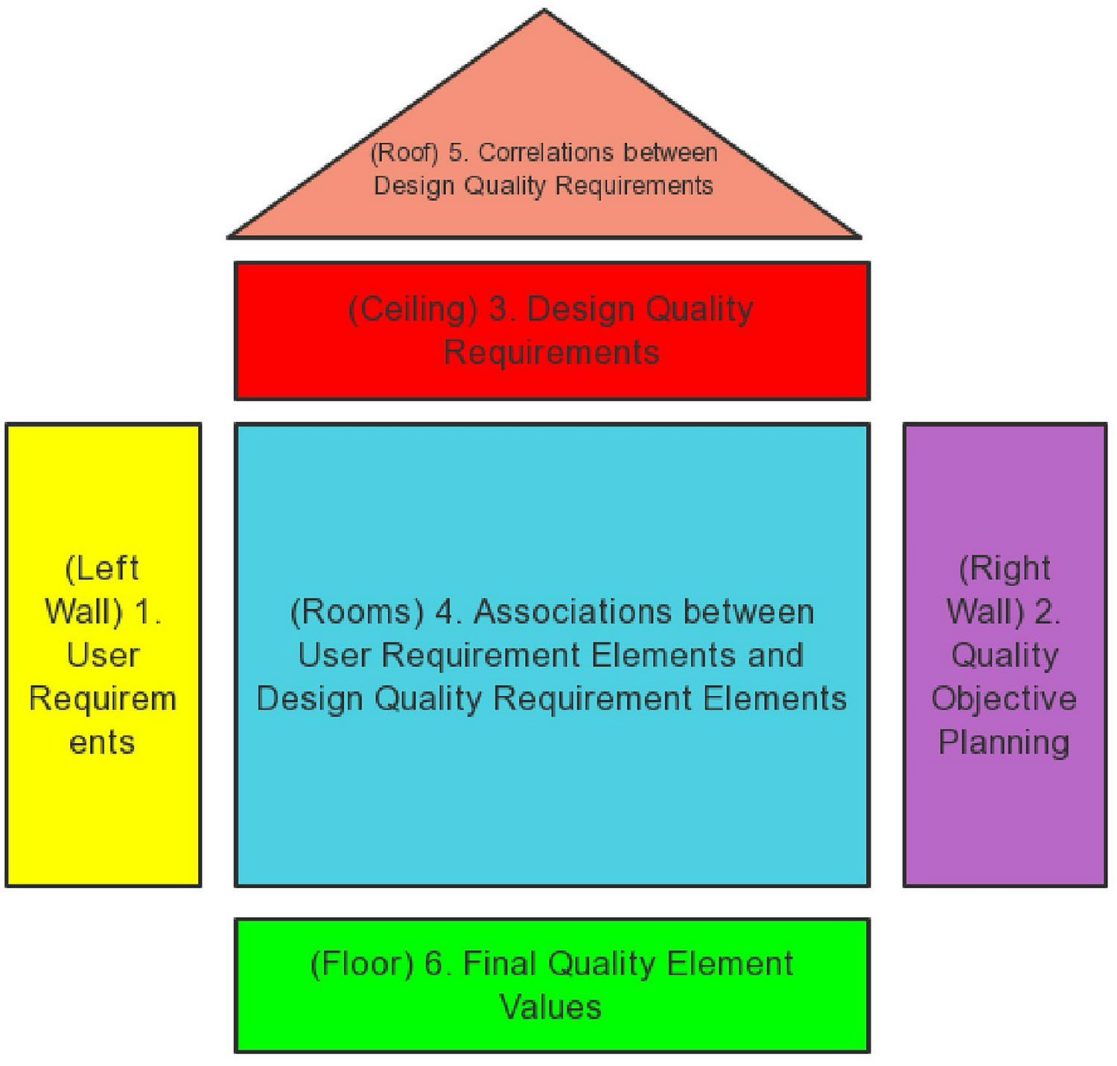
Typical QFD matrix framework.

HoQ serves as the core of QFD, illustrating the relationships among its various components, specifically, the connection between customer needs and technical requirements, thereby aiding in prioritization during the design process^56^. (Fig. 2)

In this study, determining the weight of the technical requirements for SHA technology is a crucial step in the QFD model. To achieve this, we use the relevant matrix part of QFD theory to identify and rank the key technical requirements, thereby providing a basis for subsequent design optimization and enhancing the overall performance and user satisfaction of SHA.

Once QFD has established the relationships between user needs and technical requirements, the next stage in the design process must address the uncertainty and prioritization challenges involved in implementing those requirements. At this point, the introduction of FAD becomes valuable, as it can effectively handle uncertainty in the decision-making process—particularly in cases of ambiguous or incomplete information—helping designers to make more optimal choices among multiple design alternatives for SHA.

By incorporating fuzzy mathematics into Axiomatic Design (AD), this method enables a quantitative evaluation of how different technical features affect system complexity and performance of SHA. This approach not only retains the critical needs identified through QFD but also further optimizes the design scheme, enhancing both system robustness and feasibility for SHA products.

### Kano dynamic classification mechanism

We initially quantified data obtained from the Kano model to objectively and accurately determine the initial weights of the user requirement elements. Since the I attributes in the Kano model do not affect customer satisfaction and R attributes should be excluded in the final stages of design practice, only the A, O, and M attributes were considered to precisely determine the requirement weights. To calculate the initial weights of user requirement elements, the proportions of A, O, M, and I in requirement *i* can be determined based on the Kano questionnaire results, denoted as $\:Ai$​, $\:Oi$​, $\:Mi$​, and $\:Ii$, respectively. Subsequently, the rates of satisfaction improvement, $\:{P}_{i}$​, and dissatisfaction reduction, $\:{D}_{i}$, can be calculated as

1 $$\:{P}_{i}=\frac{Ai+Oi}{Ai+Oi+Mi+Ii}$$

2 $$\:{D}_{i}=-\frac{Oi+Mi}{Ai+Oi+Mi+Ii}$$

Finally, the initial weight, $\:{\omega\:}_{i}$​, for user requirements can be obtained as

3 $$\:{\omega\:}_{i}=max(\left|{P}_{i}\right|,|{D}_{i}\left|\right)\left|\right)$$

### QFD HoQ model construction

The first step in building a product HoQ involves assembling a QFD team to conduct market research, including planning user requirements, competitive assessments, and quality targets. Competitive assessments help designers identify development directions to differentiate their products from benchmarks. Quality target planning assists designers in quantitatively analyzing quality elements, forming the basis for evaluating user needs importance in the QFD method. The initial improvement rate, $\:{IR}_{0}$, can be calculated as^50^

4 $$\:{IR}_{0}=\frac{{Q}_{b}}{{Q}_{a}}$$

where Qa and Qb are the current and target values of each requirement element, respectively. In the QFD method, adjusting IR0 can better express the importance of user requirement elements; we did this by integrating the Kano model and the QFD method. An appropriate Kano factor, k, was selected, with values for A, O, and M attributes set to 2.0, 1.0, and 0.5, respectively^57^. The revised improvement rate, $\:{IR}_{adj}$, can be calculated using the approximate transformation function as

5 $$\:{IR}_{adj}={\left({IR}_{0}\right)}^{\frac{1}{k}}$$

The adjusted quality weight, $\:{\omega\:}_{i}^{{\prime\:}}$, of user requirements can then be obtained as

6 $$\:{\omega\:}_{i}^{{\prime\:}}=\frac{{\omega\:}_{i}}{{IR}_{adj}}$$

Finally, the relative weight, $\:{\omega\:}_{i}^{{\prime\:}{\prime\:}}$​, of user requirement quality can be determined as

7 $$\:{\omega\:}_{i}^{{\prime\:}{\prime\:}}=\frac{{\omega\:}_{i}^{{\prime\:}}}{{\sum\:}_{i=1}^{m}{\omega\:}_{i}^{{\prime\:}}}$$

To transform user requirements into design quality elements and finalize design weights, a product HoQ model needs to be constructed. First, we analyzed and organized the user requirements obtained from the questionnaire. These requirements were converted into measurable and design-oriented quality elements based on measurability and correspondence criteria. Each element is related yet independent of other design requirements, as represented by the correlation matrix, *R*, expressed as.

 

8 $$R =\:\left[\begin{array}{ccccc}{R}_{11}&\:\cdots\:&\:{R}_{1j}&\:\cdots\:&\:{R}_{1n}\\\:\vdots&\:\ddots\:&\:\vdots&\:\ddots\:&\:\vdots\\\:{R}_{i1}&\:\cdots\:&\:{R}_{ij}&\:\cdots\:&\:{R}_{in}\\\:\vdots&\:\ddots\:&\:\vdots&\:\ddots\:&\:\vdots\\\:{R}_{m1}&\:\cdots\:&\:{R}_{mj}&\:\cdots\:&\:{R}_{mn}\end{array}\right]$$

where $\:{R}_{ij}$ represents the correlation between user requirement *i* and quality element *j*. Scores were assigned to each correlation matrix element by expert groups. The weights, $\:{\omega\:}_{j}$, and relative weights, $\:{\omega\:}_{j}^{{\prime\:}}$, of a quality element *j* can be calculated as

9 $$\:{\omega\:}_{j}=\sum\:_{i=1}^{m}{\omega\:}_{i}^{{\prime\:}{\prime\:}}{R}_{ij}$$

10 $$\:{\omega\:}_{j}^{{\prime\:}}={\omega\:}_{j}/\sum\:_{i=1}^{n}{\omega\:}_{j}\:$$

The final product HoQ was obtained by integrating all results obtained in this section.

### AD

**Fig. 3:**
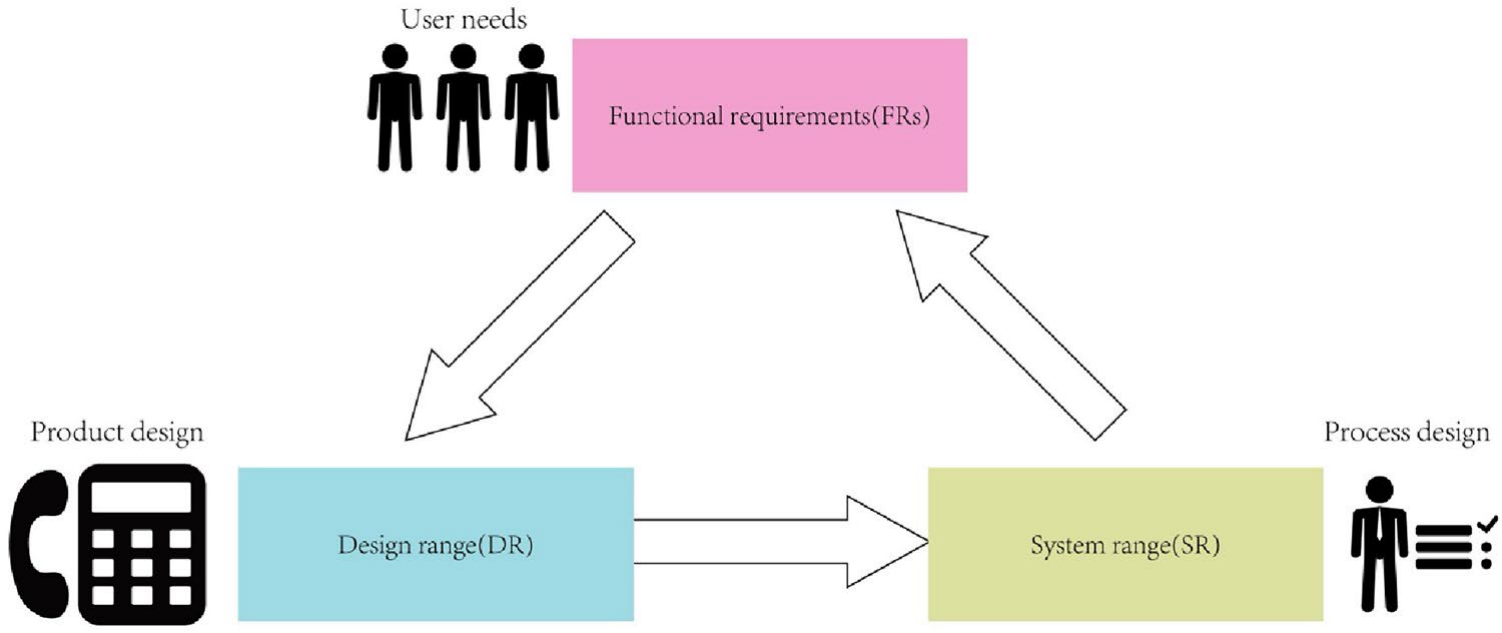
Mapping of different components within AD.

AD^4^ is a systematic and robust decision-making tool for design engineers, which aims to decompose user needs into detailed design solutions. The core process of AD involves three stages: (i) Translating user needs into specific functional requirements (FRs); (ii) selecting the most appropriate design range (DR) during the product design phase to meet the FRs; and (iii) representing the company’s capability to realize the product design as the system range (SR) in the process design phase. By establishing a mapping matrix between FRs and design range (DR), AD effectively analyzes and optimizes product designs to meet the former and improve the efficiency of existing products^58^. A certain mapping relationship exists between design elements in adjacent domains, determined by the corresponding mapping matrices (Fig. 3).

Design requirements with relatively high weights in QFD are defined and correspond to FRs in the functional domain, which are subsequently mapped to DR in the physical domain that satisfy the specific functional needs of SHA users. Subsequently, the design matrix is tested to ensure its compliance with the independence axiom. The mapping relationship between FRs and DR can be expressed as

11 $$\:\left\{FR\right\}=G\times\:DR$$

where $\:G$ is the design matrix, with $\:{G}_{ij}$​ representing the degree of association between the functional requirement $\:{FR}_{i}$​ and design parameter $\:{DR}_{j}$, i.e.,

12 $$\:{FR}_{i}=\sum\:_{j=1}^{n}{G}_{ij}\:\times\:{DR}_{j}$$

13 $$\:{G}_{ij}=\frac{\partial\:{FR}_{i}}{\partial\:{DP}_{j}}$$

where *i* = 1, 2,…,*n*; *j* = 1, 2,…, *m*; and *n* and *m* are the total number of FRs and DR, respectively.

Typically, the design matrix $\:{G}_{ij}$ can take three forms^59^:

1. *Diagonal (uncoupled design)*: $$\:\left[\begin{array}{ccc}1&\:0&\:0\\\:0&\:1&\:0\\\:0&\:0&\:1\end{array}\right]$$;
2. *Triangular (decoupled design)*: $$\:\left[\begin{array}{ccc}1&\:0&\:0\\\:1&\:1&\:0\\\:1&\:1&\:1\end{array}\right]$$;
3. *General (coupled design)*: $$\:\left[\begin{array}{ccc}1&\:1&\:1\\\:1&\:1&\:1\\\:1&\:1&\:1\end{array}\right]$$.

The design matrix $\:{G}_{ij}$ serves as a tool to represent the relationship between FRs and DR. Typically, the relationship is represented by 1 (0), indicating a strong (weak) correlation. $\:{G}_{ij}$ satisfies the independence axiom when it is a diagonal/triangular matrix, i.e., each FR can be met by a unique DR. This simplifies the design process, allowing further design studies. Conversely, a general matrix shape for *G*_*ij*_ does not satisfy the independence axiom^60^making it necessary to adjust the FRs and DR of the SHA to achieve independence.

AD is based on two important design axioms^4^:

1. Independence axiom: FRs must maintain independence.
2. Information axiom: Information content (IC) must be minimized, thereby reducing design complexity.

**Fig. 4:**
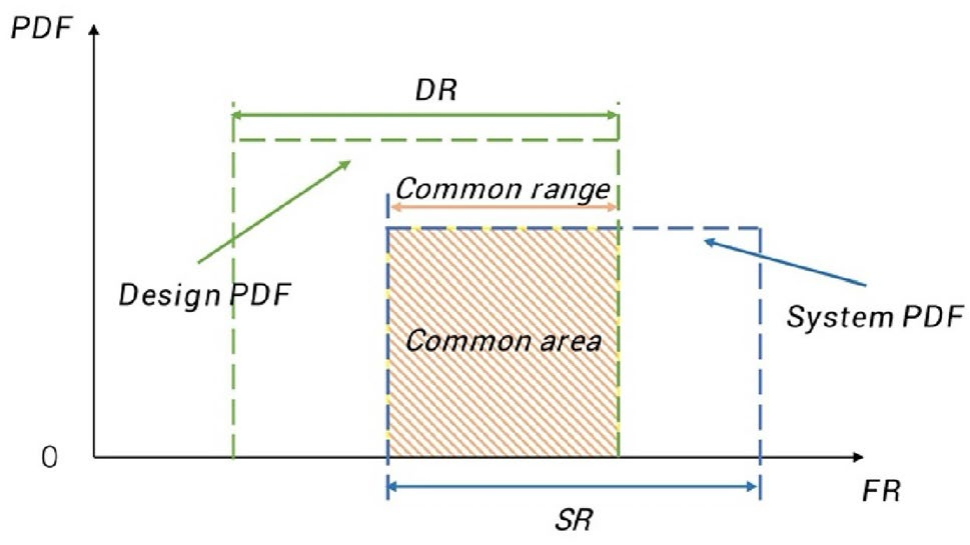
AD mapping process for a uniform FR PDF.

Designs that satisfy the Independence Axiom and minimize IC have the highest likelihood of success, making them the optimal solutions^61^. IC can be expressed as

14 $$\:IC={\text{log}}_{2}\left(\frac{1}{P}\right)$$

where *P* is the probability of meeting a given FR, which is determined by calculating the DR-SR overlapping region (common area), representing the range of acceptable solutions^8^. Specifically, DR relates to user satisfaction, while SR represents the company’s ability to meet the needs, often expressed using a probability density function (PDF)^61^. The larger the DR-SR overlap, the higher the probability of meeting the FRs. For a uniform FR PDF, the AD mapping process is illustrated in Fig. 4.

Based on the above definitions, the probability of satisfying an FR, *P*, can be expressed as

15 $$\:P=\frac{Common\:range}{SR}$$

Thus, the IC can be re-expressed as

16 $$\:IC={\text{log}}_{2}\left(\frac{SR}{Common\:range}\right)$$

In this study, the AD method is applied starting from both user needs and functional requirements. By constructing the mapping relationship between FRs and DRs, AD not only helps clarify the design logic, but also reduces coupling while maintaining the principle of system independence. This improves the rationality and feasibility of the design, thereby enhancing the system performance and structural optimization of the SHA product.

### Fuzzy AD

AD provides not only a design framework for engineers but also a means to translate user needs into technical information, ultimately creating products that satisfy users. Due to the complexity of design objects and the inherent vagueness of human thought, the relationship between FRs and DR can be fundamentally uncertain^58^. In the presence of imprecise data and fuzzy decision-making environments, data can be represented using fuzzy sets^62^. FAD addresses this issue by incorporating imprecise data into AD. In FAD, the SR and DR are expressed as TFNs, which convert the ambiguous linguistic variables used in product evaluation into numerical values via membership functions between 0 (absolute impossibility/incorrect statement) and 1 (absolute possibility/correct statement).

Assuming a TFN is represented as $\:a=({a}^{L},{a}^{M},{a}^{U})$ in the real number set $\:R$, where $\:{a}^{L}<{a}^{M}<{a}^{U}$, the membership function, $\:{\psi\:}_{a}\left(\chi\:\right)=R\to\:\left[\text{0,1}\right]$, of TFN $\:a$ can be defined as^63^

17 $$\:\psi\:\left(\chi\:\right)=\left\{\begin{array}{c}\frac{x-{a}^{L}}{{a}^{M}-{a}^{L}},{a}^{L}\le\:x\le\:{a}^{M}\\\:1,x={a}^{M}\\\:\frac{{a}^{U}-x}{{a}^{U}-{a}^{M}},{a}^{M}\le\:x\le\:{a}^{U}\\\:0,\:otherwise\end{array}\right.$$

**Fig. 5:**
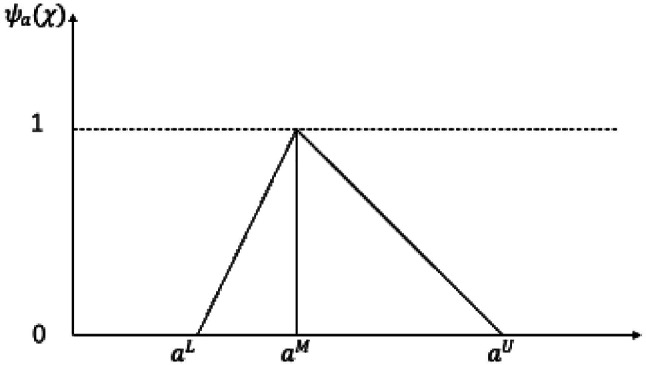
Membership function, $\:{\psi\:}_{a}\left(\chi\:\right)$, of a TFN.

The membership function, $\:{\psi\:}_{a}\left(\chi\:\right)$, of a TFN is defined across different intervals (Fig. 5). The membership range is [0,1], where $\:{a}^{L}$, $\:{a}^{M}$, and $\:{a}^{U}$ are the lower boundary, middle value, and upper boundary, respectively, corresponding to the minimum, median, and maximum values of the evaluation score.

When $\:{a}^{L}<x<{a}^{M}$, the membership function $\:{\psi\:}_{a}\left(\chi\:\right)$ increases linearly as $\:x$ increases, indicating gradual strengthening of membership as the design parameter approaches the median value. At $\:x={a}^{M}$, the membership degree reaches its maximum of 1, suggesting $\:{a}^{M}$ to be the optimal/most likely design parameter. For $\:{a}^{M}<x<{a}^{U}$, $\:{\psi\:}_{a}\left(\chi\:\right)$ decreases linearly from 1 to 0 as $\:x$ decreases from $\:{a}^{M}$ to $\:{a}^{U}$. This reflects that as the design parameter deviates from $\:{a}^{M}$, its membership in the fuzzy set weakens. For $\:x<{a}^{L}$ or $\:x>{a}^{U}$, the membership degree is 0, indicating that parameters outside this range do not satisfy the requirements.

The SR and corresponding DR were normalized through an expert score, $\:x$, as

18 $$\:\mathcal{Z}=\frac{x-min\left(x\right)}{max\left(x\right)-min\left(x\right)}$$

to define the fuzzy system range (FSR) and fuzzy design range (FDR). If the FSR is represented as $\:\left({x}_{a},{x}_{b},{x}_{c}\right)$, where $\:{x}_{a}$​, *x*_*b*_, and $\:{x}_{c}$​ are the minimum, median, and maximum values, respectively, and the FDR is represented as $\:\left({x}_{k},{x}_{d},{x}_{d}\right)$, where $\:{x}_{k}$ are the minimum, and *x*_*d*_ are the median and maximum values, respectively, their membership degrees are both equal to 1.

In FAD, expert evaluations of design range must be converted into TFNs to quantify the vagueness and uncertainty inherent in subjective assessments. The specific conversion process is as follows:

1. Suppose five experts rate the same function as 4, 5, 6, 7, and 8 (on a 1–9 scale). According to Eq. 17, the triangular fuzzy number for Expert 1’s evaluation is $\:\left({a}^{L},{a}^{M},{a}^{U}\right)$ = (3, 4, 5).

$$\:{\psi\:}_{1}\left(\chi\:\right)=\left\{\begin{array}{c}\frac{x-{a}^{L}}{{a}^{M}-{a}^{L}},3\le\:x\le\:5\\\:1,x=4\\\:\frac{5-x}{5-4},4\le\:x\le\:5\\\:0,\:otherwise\end{array}\right.$$

Similarly, the membership functions for Expert 2 (4, 5, 6) and Expert 3 (5, 6, 7) can be defined accordingly.

2. Calculate the average of all experts’ fuzzy numbers:

$$\:\left(\frac{3+4+5}{3},\frac{4+5+6}{3},\frac{5+6+7}{3}\right)=(4,5,6)$$

3. According to Eq. 18, the Triangular Fuzzy Number representing expert scores for the design parameter, FDR$\:\left({x}_{k},{x}_{d},{x}_{x}\right)$, is:

$$\:\mathcal{Z}=(\frac{4-1}{8},\frac{5-1}{8},\frac{6-1}{8})=(0.375,0.5,0.625)$$

The Better (B) and Worse (W) coefficients derived from the KANO model must also be converted into TFNs to quantify the vagueness and uncertainty of subjective evaluations. The specific conversion rules are as follows:

The Kano coefficients are first linearly transformed from the interval [− 1, 1] to [0, 1]. The left endpoint ($\:{a}^{L}$) is determined by the Worse coefficient, representing the minimum potential negative impact of user dissatisfaction:

19 $$\:{x}_{k}=\left(\frac{1+{D}_{i}}{2}\right)$$

It is assumed that there is a “ceiling effect” on user satisfaction improvement—once a product possesses a specific functional attribute, user satisfaction approaches its theoretical maximum (i.e., approaching 1), and further enhancement yields minimal perceptual benefit. Under this assumption, both the middle point ($\:{a}^{M}$) and right endpoint ($\:{a}^{U}$) are determined by the Better coefficient, representing the core positive impact on user satisfaction:

20 $$\:{a}^{M}={a}^{U}=\left(\frac{1+{P}_{i}}{2}\right)$$

Thus, the FDR is defined as:

 

21 $$\:\left({x}_{k},{x}_{d},{x}_{d}\right)=\:\left(\frac{1+{D}_{i}}{2},\frac{1+{P}_{i}}{2},\frac{1+{P}_{i}}{2}\right)$$

**Fig. 6:**
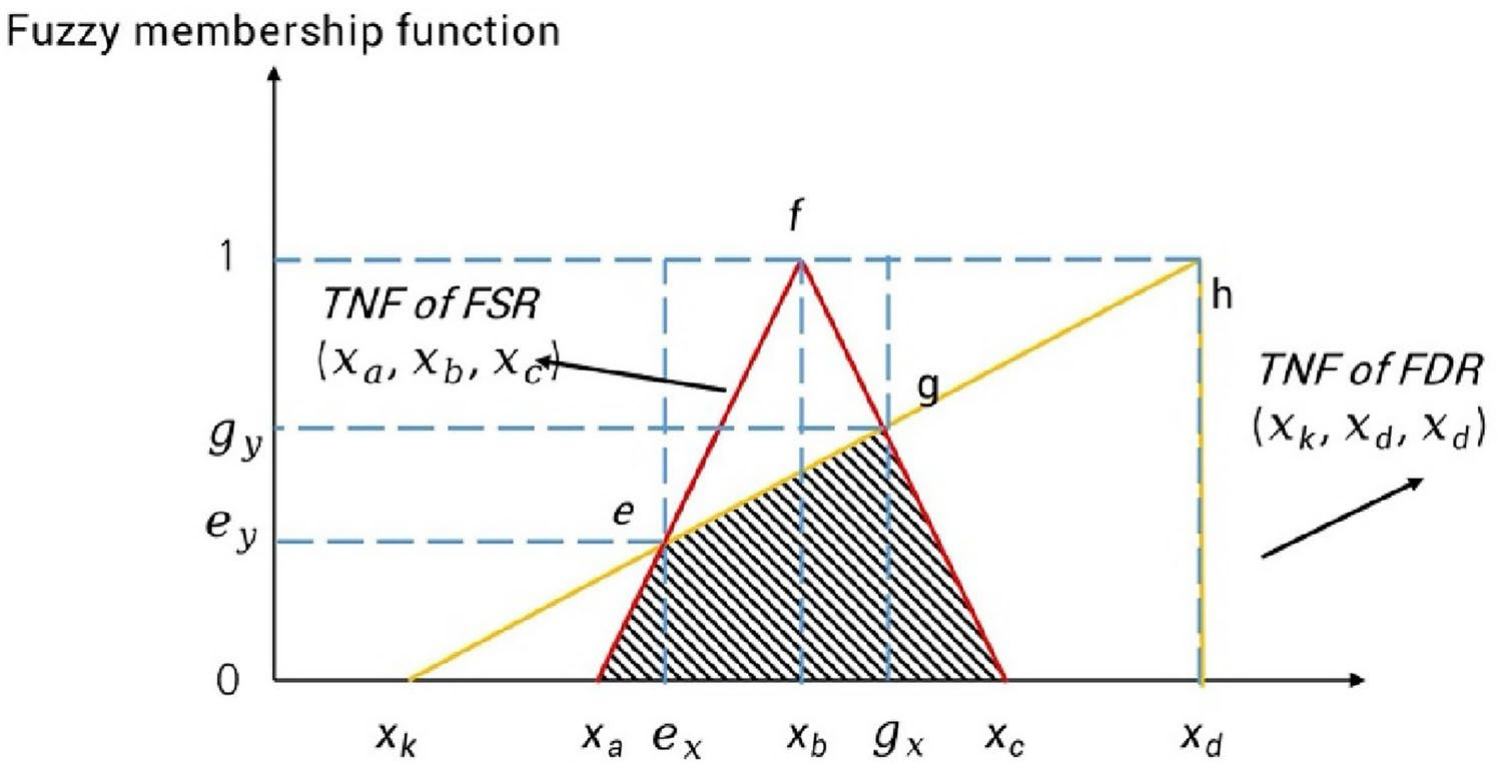
FSR-FDR TFN overlapping region.

Through the normalization of expert scores, and based on Eqs. (18–21), the Kano model’s better-worse coefficient can be mapped to the same interval as the FSR, i.e., (0, 1). The overlap between the FSR and FDR can then be analyzed to determine the probability of meeting the design requirements. The FSR-FDR overlapping area represents the degree to which the design solution satisfies the FRs (Fig. 6); the larger the overlapping area, the more the Functional requirements meet the FRs.

Thus, the IC in FAD can be defined as^64^

22 $$\:IC={\text{log}}_{2}\left(\frac{TFN\:of\:FSR}{Common\:area}\right)$$

The area of the TFNs of the FSR can be expressed as

23 $$\:TFN\:of\:FSR=\frac{1}{2}\left|{x}_{a}-{x}_{c}\right|$$

When the coordinates of the triangle vertices are known, the intersection points $\:e$ and $\:g$ can be calculated using the vector method. Based on Fig. 5, the endpoints of the line segments can be obtained as follows:

1. For $$\:{x}_{a}f$$​: $$\:\left({x}_{a},0\right)$$ and $$\:\left({x}_{b},1\right)$$.
2. For $$\:{x}_{k}h$$​:$$({x}_{k},0)$$and $$\:\left({x}_{d},1\right)$$.
3. For $$\:\overline{f{x}_{c}}$$​​: $$\:\left({x}_{b},1\right)$$ and $$\:\left({x}_{c},0\right)$$.

The parametric equations for vector $\:r$ are expressed in Eqs. (24), (25), and (26):

24 $$\:r\left(\overline{{x}_{a}f}\right)=\left({x}_{a}+{t}_{a}\left({x}_{b}-{x}_{a}\right),{t}_{a}\right),\:{t}_{a}\in\:\left[0,1\right]$$

25 $$\:r\left(\overline{{x}_{k}h}\right)=\left({x}_{k}+{t}_{b}\left({x}_{d}-{x}_{k}\right), {t}_{b}\right),\:{t}_{b}\in\:\left[0,1\right]$$

26 $$\:r\left(\overline{f{x}_{c}}\right)=\left({x}_{b}+{t}_{c}\left({x}_{c}-{x}_{b}\right),1-{t}_{c}\right),\:{t}_{b}\in\:\left[0,1\right]$$

An intersection point occurs when two line segments have the same coordinates in their parametric equations. Thus, the following conditions must be satisfied:

1. *When*$$\:\:\overline{{x}_{a}f}$$ *intersects* $$\:\overline{{x}_{k}h}$$: $$\:{x}_{a}+{t}_{a}\left({x}_{b}-{x}_{a}\right)={x}_{k}+{t}_{b}\left({x}_{d}-{x}_{k}\right)$$, $$\:{t}_{a}={t}_{b}$$
2. *When* $$\:\overline{{x}_{k}h}$$ *intersects* $$\:\overline{f{x}_{c}}$$: $$\:{x}_{k}+{t}_{b}\left({x}_{d}-{x}_{k}\right)={x}_{b}+{t}_{c}\left({x}_{c}-{x}_{b}\right)$$, $$\:{t}_{b}$$*=*$$\:1-{t}_{c}$$.

The simplified equations can be then expressed as:

27 $$When\;\:{t}_{a}\;intersects\; \:{t}_{b}:\:{t}_{a}={t}_{b}=\frac{{x}_{a}-{x}_{k}}{{x}_{a}-{x}_{b}+{x}_{d}-{x}_{k}}$$

 

28 $$When\;\:{t}_{b}\;intersects\; \:{t}_{c}:\:{t}_{c}=\frac{{x}_{b}-{x}_{d}}{{x}_{b}-{x}_{c}-{x}_{d}+{x}_{k}},\:{t}_{b}=\frac{-{x}_{c}+{x}_{k}}{{x}_{b}-{x}_{c}-{x}_{d}+{x}_{k}}$$

The common area is represented by the right trapezoid $\:{x}_{1}{x}_{2}eg$, combined with the areas of triangles $\:{x}_{1}{x}_{a}e$ and $\:{x}_{2}{x}_{c}g$ (Fig. 5), which can be obtained as

29 $$\:Common\:area=\frac{1}{2}\left({e}_{y}+{g}_{y}\right)\left({x}_{c}-{x}_{a}\right)+\frac{1}{2}{e}_{y}\left({x}_{1}-{x}_{a}\right)+\frac{1}{2}{g}_{y}\left({x}_{c}-{x}_{2}\right)$$

## Two-stage framework design

The evaluation framework in this study was based on 5 main criteria and 17 sub-criteria. To assess user satisfaction, 20 evaluation standards were established, and a questionnaire was designed using the Kano model to determine user satisfaction levels. Subsequently, the QFD method was employed to translate customer needs into technical requirements. Equations (4–10) were used to calculate the values of each criterion, guiding the design range that meet SHA user needs. These parameters also significantly reduced sampling uncertainties.

After determining the design range, the better-worse coefficients of the Kano model were used to calculate the FDR and FSR for each design parameter. Subsequently, Eq. (25) was applied to compute the overlap between FDR and FSR, which in turn provided the IC of each criterion. The IC value was then calculated using Eq. (19). The criteria were ranked based on IC values; smaller IC values indicate a smaller gap between the user and the company, while larger values suggest that the criterion should be prioritized for improvement. This process identifies the strengths/weaknesses of the product to optimize company resource allocation.

**Fig. 7:**
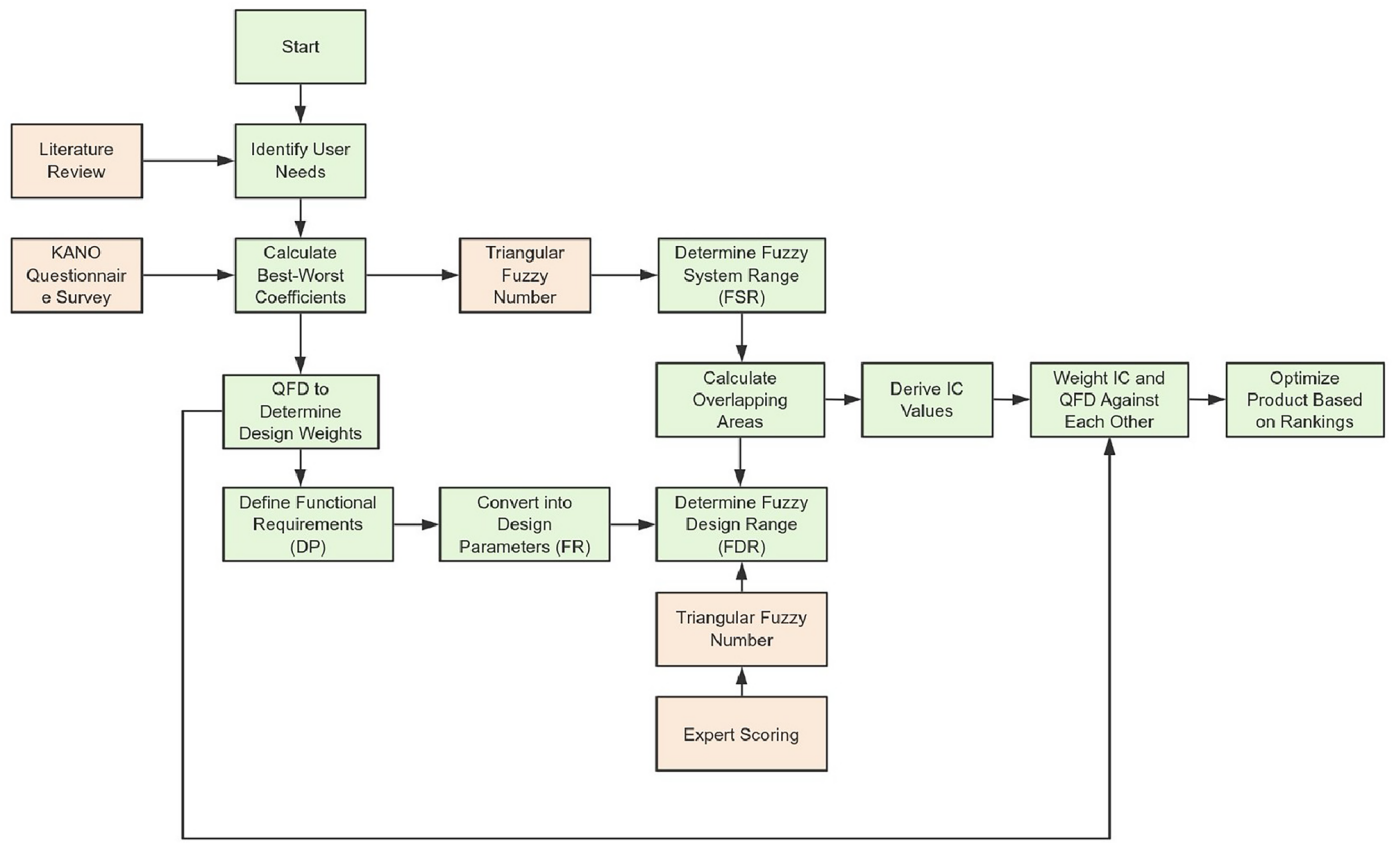
Schematic flow of the proposed Kano-QFD + FAD method.

Finally, the quality weights derived from QFD were applied to the IC values. Specifically, the quality weight reflects the relative importance of each criterion compared to others. This process ensures prioritization of criteria with larger IC values while considering the importance of user needs. By weighting the IC values for each criterion, a composite ranking was calculated, enabling designers to identify the most critical areas for improvement. Finally, product requirements were ranked based on the weighted IC values, with smaller IC values indicating the criteria that are more crucial to meeting user needs. This provides a clear basis for the optimal product design solution (Fig. 7).

## Case study

Based on the previously proposed two-stage Kano-QFD-FAD integrated framework, this study selects three mainstream SHAs available on the market as empirical subjects to verify the practical applicability of the framework and to identify the priority of user needs. The selected products (see Table 4) represent the three core design directions currently guiding the development of SHAs:

**Table 4:** Comparison of core performance parameters of three mainstream senior hearing aid products.

	Product A	Product B	Product C
Type	In-ear	Behind-the-ear	Bone conduction
Weight (g)	6.7	12	27
Noise Reduction (dB SNR)	30	$\:\le\:$29 + 3	64
Battery Life (hours)	19	40	8

Note: The noise reduction performance of Product B is marked as “≤29 + 3 dB,” indicating that its maximum noise reduction under adaptive mode is 32 dB.

1. Lightweight design and noise reduction performance optimization [Product A: in-ear type, dB SNR (Signal-to-Noise Ratio), lightweight];
2. Adaptive scene adjustment [Product B: behind-the-ear type, supports multiple environmental modes, long battery life];
3. Bone conduction and smart connectivity [Product C: bone conduction type, bone conduction gain].

To demonstrate the implementation of the proposed Kano-QFD + FAD method, we conducted a survey targeting consumers aged ≥ 60 y. This user research used a combination of interviews and questionnaire surveys and engaged a total of 321participants.

The questionnaire was based on the principles of the Kano model, aiming to refine and classify user requirements for SHA design. The questionnaire included paired questions addressing both positive and negative aspects of each product feature. For example, corresponding positive and negative questions addressing earphone wearing comfort can be as follows: *“Do you feel that comfortable earphones enhance your user experience?”* and *“Would you feel inconvenienced if the earphones were uncomfortable to wear?”*. This approach enables a comprehensive understanding of users’ perceptions of hearing aids. To ensure its validity, the questionnaire design was based on existing research, subjected to expert review, and pre-tested on a small scale to verify its reliability and rationality.

The questionnaire covered five levels of user needs defined in the Kano model, namely *“Very Attractive”*, *“Must be”*, *“Indifferent”*, *“Acceptable”*, and *“Not Attractive”*, to capture users’ perceptions across various demand elements. The questionnaire was distributed online, allowing participants to easily complete and submit their responses (see Table 5). A total of 329 questionnaires were distributed, and 321 valid responses were received, representing a response rate of 97.6%. These 321 participants formed the sample for this case study. To enhance the reliability and representativeness of the findings, participants’ gender, participants’ age distribution, and Hearing-related product usage are detailed below. Specifically, among all the participants, 55.76% were male, and 44.24% were female. And 25.86% were aged 60–69, 33.64% aged 70–79, 26.79% aged 80–89, and 13.71% were aged 90 and above. Among the elderly participants (aged ≥ 60), 65.42% regularly used hearing aids in daily life, while the remaining 34.58% did not use any assistive hearing devices (see Table 6). The eight unreturned questionnaires were likely due to network issues or incomplete submissions. Despite this small number of uncollected responses, the high response rate ensured the overall representativeness and reliability of the data(see Table 7). Details of the questionnaire are provided in Appendix1.

**Table 5:** Kano questionnaire.

Question	Does the elderly hearing aid have good tactile feedback?	Very attractive	Must be	Indifferent	Acceptable	Not attractive
*If this feature is present*,* how would you rate it?*		5	4	3	2	1
*If this feature is absent*,* how would you rate it?*		5	4	3	2	1

**Table 6:** Demographic characteristics of participants (*n* = 321).

Item	Category	Percent (%)
Gender	Male	55.76%
	Female	44.24%
Age	60–69 years old	25.86%
	70–79 years old	33.64%
	80–89 years old	26.79%
	90 years old and above	13.71%
Hearing aid usage	Yes	65.42%
	No	34.58%

**Table 7:** Analysis of Kano model results.

Feature	Kano attribute	Better coefficient	Worse coefficient
C14	*M*	32.18%	−51.1%
C32	*M*	30.35%	−47.6%
C11	*M*	26.54%	−55.34%
C21	*O*	57.55%	−50.94%
C31	*O*	54.95%	−45.69%
C12	*O*	52.75%	−49.84%
C53	*O*	52.55%	−51.91%
C22	*A*	51.42%	−27.13%
C51	*A*	50.47%	−19.87%
C33	*A*	50.16%	−25.55%
C34	*A*	49.69%	−27.04%
C13	*A*	48.74%	−27.99%
C42	*A*	48.58%	−26.18%
C41	*I*	33.12%	−29.97%
C43	*I*	32.08%	−31.45%
C52	*I*	29.02%	−25.87%
C23	*I*	27.51%	−26.54%

In addition, reliability and validity tests indicated that the questionnaire data were suitable for further analysis. Cronbach’s α coefficients were 0.819 and 0.826, the KMO value was 0.864 (> 0.6), and Bartlett’s test of sphericity was significant at *p* < 0.001, demonstrating high reliability and suitability for factor analysis. Detailed results of the reliability and validity tests are provided in the Appendix2.

Based on the results, we excluded 4 I attributes and identified 13 key requirements for SHAs, and classified them into three categories:

1. *Basic needs: Comfortable earplug design (C14)*,* high sound quality (C32)*,* and lightweight product (C11)*.
2. *Expected needs: Color design (C21)*,* simple button operation (C31)*,* good tactile feedback (C12)*,* and low maintenance cost (C53)*.
3. *Attractive needs: Creative design (C22)*,* high cost–performance ratio (C51)*,* long-lasting battery life (C33)*,* adaptability to different environments (C34)*,* compact size (C13)*,* and internal structure (C42)*.

Quality objective planning is a social survey based on a demand index system, focusing on the evaluation the three SHAs products investigated in Table 4: Product A, Product B, and Product C. In this study, the competitive scale for quality objective planning was classified into five levels: *“Very strong”*, *“Strong”*, *“Average”*, *“Weak”*, and *“Very weak”*, corresponding to the scores of 5, 4, 3, 2, and 1, respectively (see Table 8).

**Table 8:** Quality objective planning.

User demand elements	Demand	Competitive evaluation			Current value (Q_a_)	Planning quality objectives (Q_b_)
	type	Product A	Product B	Product C		
C11	*M*	4	3	3	3	4
C12	*O*	2	1	3	2	4
C13	*A*	3	2	3	3	4
C14	*M*	2	2	3	2	3
C21	*O*	1	2	2	2	3
C22	*A*	2	3	3	3	3
C31	*O*	4	2	5	4	5
C32	*M*	2	1	3	2	3
C33	*A*	2	3	2	2	4
C34	*A*	2	2	3	2	3
C42	*A*	2	2	5	3	4
C51	*A*	3	4	1	3	3
C53	*O*	2	3	2	2	3

The competitiveness scores for Product A and Product B were relatively similar across the majority of demand elements but were generally lower than those for Product C. This was especially evident for high-priority demands, such as C31 and C42, where Product C demonstrated superior performance. Additionally, several demand elements showed a significant gap between *Q*_*a*_ and *Q*_*b*_, indicating areas requiring improvement. Notably, C12, C11, and C42 showed significantly lower *Q*_*a*_ than *Q*_*b*_, highlighting substantial potential for their enhancement. Improving these demand elements is crucial for boosting product competitiveness.

To further optimize product design and reduce the gap between *Q*_*a*_ and *Q*_*b*_, we established a correlation matrix between user demand elements and quality elements. Correlations between the two elements were scored from 9, 7, 5, 3, and 1 (to indicate relationships from strong to weak) by industry experts, university professors, and practitioners. A lack of numerical score indicates no relationship between user demand and quality elements. The relative weights of product elements were calculated using the scoring data, resulting in a comprehensive product HoQ (see Table 9).

**Table 9:** HoQ for SHAs.

User demand elements	H1	H2	H3	H4	H5	H6	H7	H8	H9	H10	H11	H12	H13	$\:{\omega\:}_{i}$	$\:{IR}_{0}$	k	$\:{IR}_{adj}$	$\:{\omega\:}_{i}^{{\prime\:}}$	$\:{\omega\:}_{i}^{{\prime\:}{\prime\:}}/\text{\%}$
C11	9	3	7	3			1		9		5	3	1	0.5534	1.33	0.5	1.7689	0.3128	6.5
C12	7	9		7	3				3				3	0.5275	2	1	2	0.2638	5.48
C13	1	3	9	3		3	1		7		7		3	0.4874	1.33	2	1.1533	0.4226	8.78
C14	5	5	1	9		3		3		3				0.5110	1.5	0.5	2.25	0.2271	4.72
C21		3			9	3								0.5755	1.5	1	1.5	0.3837	7.97
C22	3		3		7	9					5	3		0.5142	1	2	1	0.5142	10.69
C31							9					1	1	0.5495	1.25	1	1.25	0.4396	9.13
C32								9		7		1	3	0.4760	1.5	0.5	2.25	0.2116	4.4
C33			5					3	9	3	1	3	3	0.5016	2	2	1.4142	0.3547	7.37
C34				3			1	7		9		1	1	0.4969	1.5	2	1.2247	0.4057	8.43
C42	9	3	7	1		5	1		1		9		7	0.4858	1.33	2	1.1533	0.4211	8.75
C51	3	1		1		1		3	5			9	5	0.5047	1	2	1	0.5047	10.49
C53	1		5					1			7	1	9	0.5255	1.5	1	1.5	0.3503	7.28
$\:{\omega\:}_{j}$	278.81	179.41	295.81	171.21	163	214.86	114.63	173.63	263.93	142.94	284.49	197.33	246.37						
$\:{\omega\:}_{j}^{{\prime\:}}$/%	10.23	6.58	10.85	6.28	5.98	7.88	4.20	6.37	9.68	5.24	10.43	7.24	9.04						

Note: H1: selection of lightweight, durable, and high-quality tactile materials; H2: casing material and surface treatment technology; H3: miniaturized component design and battery size optimization; H4: earbud comfort design and material selection; H5: coating material and color processing; H6: industrial design and ergonomic design; H7: button design and control interface design; H8: sound processing technology and microphone/speaker quality; H9: battery optimization and low-power design; H10: automatic adjustment function and personalized customization; H11: optimized structural design; H12: cost control and pricing strategy; and H13: maintenance-friendly design and easy-to-replace components.

**Fig. 8:**
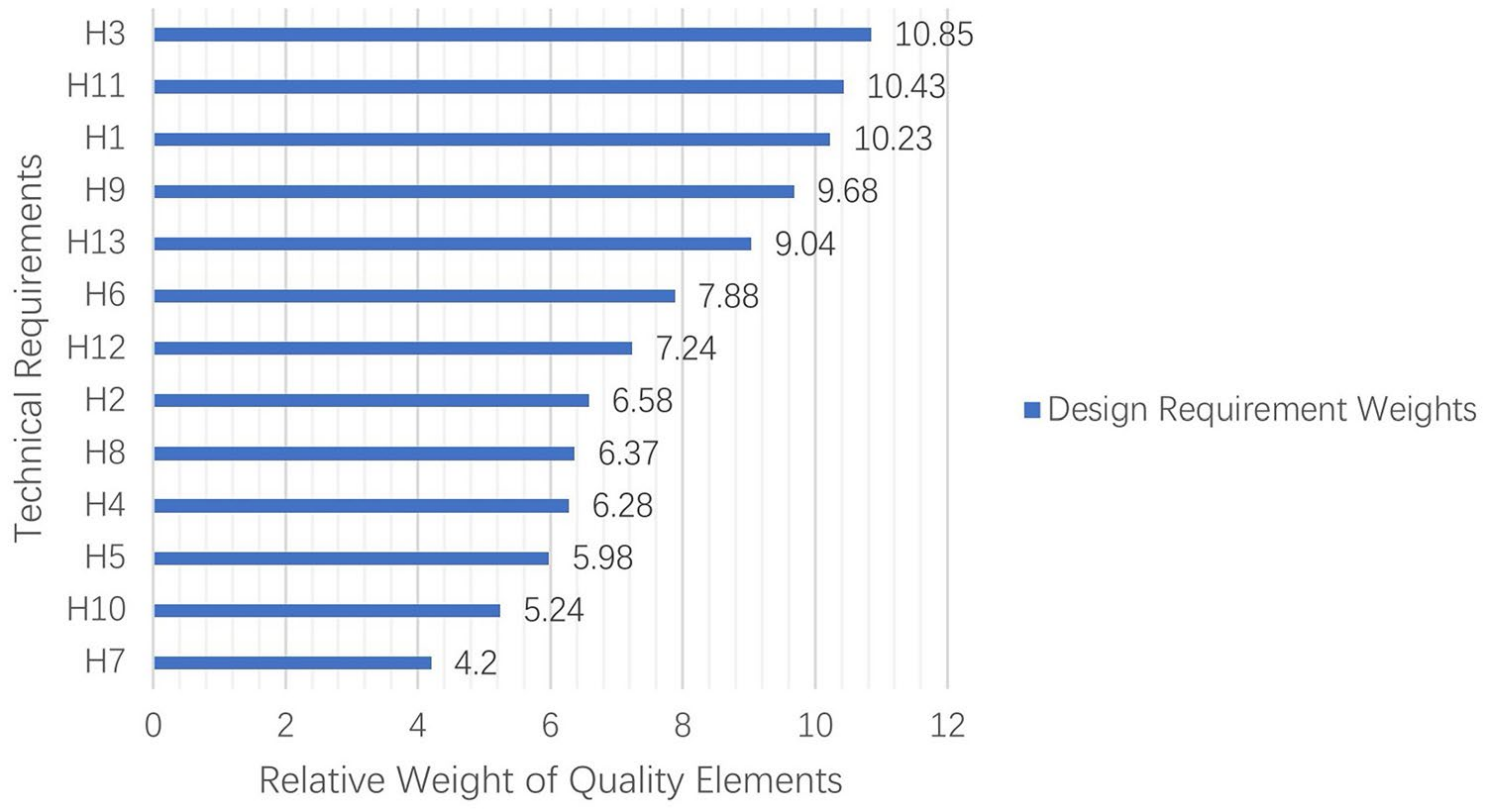
Priority weight distribution of design requirements.

Figure 8 shows the normalized priority rankings of design requirements, representing their corresponding priority weights. A functional demand model was subsequently established for the SHAs.

We selected the most critical design requirements obtained from the QFD analysis as functional demands for SHAs. Thereafter, we integrated the design recommendations from 20 experts to formulate specific DR to meet these functional demands (see Table 10). These 20 experts were unequally divided into three groups. The selection criteria for the experts included: (1) more than 10 years of experience in design or related fields; (2) recognized influence in academia or industry; and (3) representation from different institutions to ensure diversity of perspectives. Subsequently, each DR was scored on a scale of 1–9 by groups of experts. Each group then engaged in internal discussion and opinion exchange regarding their members’ scoring results. Based on thorough communication, scores with significant differences were adjusted, and a unified score was finalized at the group level. Next, we aggregated the scoring results from the three groups. For items with large scoring discrepancies (i.e., scores from a group deviating from the overall mean by more than one standard deviation), we organized online discussions to invite experts to clarify and explain these differences. If necessary, experts revised their scores based on the outcomes of the discussions. The final scores were obtained by averaging the scores from all experts. The scores were standardized and incorporated with the Kano model data to obtain the FDR and FSR. These fuzzy values were expressed using TFNs (see Table 11).

**Table 10:** Comparison of FR and DR for SHAs.

Technical requirements (H)	Design range (DR)	Functional requirements (FR)
H5 and H6	$\:{DR}_{1}$: aesthetic design	$\:{FR}_{1}:$ modern aesthetic design language
H1 and H2	$\:{DR}_{2}:$ material process	$\:{FR}_{2}$: durable high-strength plastics
H11	$\:{DR}_{3}$: portability	$\:{FR}_{3}$: lightweight structure
H3 and H9	$\:{DR}_{4}$: long battery life	$\:{FR}_{4}$: high-capacity battery and battery management system
H12	$\:{DR}_{5}:$ high cost performance	$\:{FR}_{5}$: modular design
H4 and H8	$\:{DR}_{6}$: clear sound quality	$\:{FR}_{6}:$ audio components and acoustic design
H10	$\:{DR}_{7}:$ high adaptability	$\:{FR}_{7}$: environmental mode audio noise reduction
H7	$\:{DR}_{8}$: easy operation	$\:{FR}_{8}$: simple button interaction
H13	$\:{DR}_{9}:$ easy maintenance	$\:{FR}_{9}$: easy-to-remove components

**Table 11:** FDR and FSR scoring.

	Expert Group 1	Expert Group 2	Expert Group 3	FDR $\:\left({x}_{k},{x}_{d},{x}_{d}\right)$	FSR $\:\left({x}_{a},{x}_{b},{x}_{c}\right)$
$\:{FR}_{1}$	5	7	6	(0.305, 0.772, 0.772)	(0.500, 0.625, 0.750)
$\:{FR}_{2}$	6	5	5	(0.237, 0.698, 0.698)	(0.416, 0.541, 0.666)
$\:{FR}_{3}$	5	5	7	(0.369, 0.743, 0.743)	(0.458, 0.583, 0.708)
$\:{FR}_{4}$	5	7	5	(0.366, 0.747, 0.747)	(0.458, 0.583, 0.708)
$\:{FR}_{5}$	6	4	7	(0.401, 0.752, 0.752)	(0.458, 0.583, 0.708)
$\:{FR}_{6}$	7	5	3	(0.253, 0.656, 0.656)	(0.375, 0.500, 0.625)
$\:{FR}_{7}$	7	4	4	(0.365, 0.748, 0.748)	(0.375, 0.500, 0.625)
$\:{FR}_{8}$	6	6	6	(0.272, 0.775, 0.775)	(0.500, 0.625, 0.750)
$\:{FR}_{9}$	4	7	7	(0.240, 0.763, 0.763)	(0.500, 0.625, 0.750)

During the merging of certain quality elements (H) into a single FR, the corresponding DR were determined by averaging the ‘better’ or ‘worse’ coefficients from the Kano model for the two merged requirements. Similarly, the quality weights in the QFD analysis were derived by averaging these values. Analysis of the overlap between the FDR and FSR revealed that some FRs, such as $\:{FR}_{1}$ and $\:{FR}_{8}$, aligned well with the systemic requirements, with median values being close and requiring only minor adjustments. However, other requirements, such as $\:{FR}_{2}$, $\:{FR}_{3}$, $\:{FR}_{4}$, $\:{FR}_{5}$, $\:{FR}_{6}$, $\:{FR}_{7}$, and $\:{FR}_{9}$, exhibited significant discrepancies between their DR and systemic requirements. Notably, $\:{FR}_{3}$, $\:{FR}_{5}$, and $\:{FR}_{9}$ showed substantial differences in their median values, indicating a need for considerable optimization. Therefore, further adjustments are necessary to ensure consistency between the design and system requirements. Overall, refining these design range will enhance the alignment between the product and system demands, leading to SHA design optimization.

In the design process, the relationships between the FRs and DR were represented using a mapping relationship matrix, *G*, derived using Eqs. (7–9) as

$$\:G=\left[\begin{array}{c}{FR}_{1}\\\:{FR}_{2}\\\:\begin{array}{c}{FR}_{3}\\\:{FR}_{4}\\\:\begin{array}{c}{FR}_{5}\\\:{FR}_{6}\\\:{FR}_{7}\\\:{FR}_{8}\\\:{FR}_{9}\end{array}\end{array}\end{array}\right]=\left[\begin{array}{ccccccccc}1&\:0&\:0&\:0&\:0&\:0&\:0&\:0&\:0\\\:0&\:1&\:0&\:0&\:0&\:0&\:0&\:0&\:0\\\:0&\:0&\:1&\:0&\:0&\:0&\:0&\:0&\:0\\\:0&\:0&\:0&\:1&\:0&\:0&\:0&\:0&\:0\\\:0&\:0&\:0&\:0&\:1&\:0&\:0&\:0&\:0\\\:0&\:0&\:0&\:0&\:0&\:1&\:0&\:0&\:0\\\:0&\:0&\:0&\:0&\:0&\:0&\:1&\:0&\:0\\\:0&\:0&\:0&\:0&\:0&\:0&\:0&\:1&\:0\\\:0&\:0&\:0&\:0&\:0&\:0&\:0&\:0&\:1\end{array}\right]=\left[\begin{array}{c}{DR}_{1}\\\:{DR}_{2}\\\:\begin{array}{c}{DR}_{3}\\\:{DR}_{4}\\\:\begin{array}{c}{DR}_{5}\\\:{DR}_{6}\\\:{DR}_{7}\\\:{DR}_{8}\\\:{DR}_{9}\end{array}\end{array}\end{array}\right]$$

In AD theory, the diagonal design matrix *G* represents a decoupled design that adheres to the independence axiom. This compliance ensures the independence of DR within the system, avoiding complex interferences or correlations. Consequently, the structure of the design matrix indicates logical soundness in the proposed DR, providing theoretical support and a foundation for the design of SHAs.

By establishing a clear mapping relationship, the designers can identify the corresponding DR of each FR, enabling targeted optimization and improvement. This approach ensures the independence of various functions and enhances the efficiency of the design process. The FSR area and the coordinates of the intersection points for each requirement were calculated using Eqs. (17–18). The overlapping area between the FDR and FSR was then determined based on the obtained coordinates using Eqs. (23–29) (see Table 12).

**Table 12:** TFN of FSR and common areas.

	TFN of FSR	$\:Common\:area$
$\:{FR}_{1}$($\:{DR}_{1}$)	0.1250	0.1110
$\:{FR}_{2}$($\:{DR}_{2}$)	0.1250	0.1903
($\:{\text{F}\text{R}}_{3}{\text{D}\text{R}}_{3}$)	0.1250	0.0992
$\:{FR}_{4}$($\:{DR}_{4}$)	0.1250	0.0990
$\:{FR}_{5}$($\:{DR}_{5}$)	0.1250	0.0918
$\:{FR}_{6}$($\:{DR}_{6}$)	0.1250	0.1043
$\:{FR}_{7}({DR}_{7})$	0.1250	0.0663
$\:{FR}_{8}$($\:{DR}_{8}$)	0.1250	0.1132
$\:{FR}_{9}({DR}_{9})$	0.1250	0.1158

The IC value was calculated using Eq. (22). A smaller IC value indicates that the design aligns more closely with user expectations. Conversely, a larger IC value signifies greater discrepancies in the design dimension, suggesting a greater need for improvement. The IC values were then weighted by the QFD-derived quality weights; as a higher QFD weight corresponds to a greater importance of the requirement to the user. Consequently, the final improvement priorities were established, and the DR were ranked to determine the optimal design solution (see Table 13). To verify the rationality of the QFD-derived weights, a sensitivity analysis was conducted on the IC ranking results. When different weight assignment strategies were applied (e.g., equal weighting or expert-average weighting), the rankings of the main demand items remained consistent, indicating strong robustness of the results.

**Table 13:** Final FR rankings.

	IC	Final results	Ranking
$\:{FR}_{1}$($\:{DR}_{1}$)	0.1544	0.0107	7
$\:{FR}_{2}$($\:{DR}_{2}$)	0.1947	0.0164	6
$\:{FR}_{3}({\text{D}\text{R}}_{3})$	0.3219	0.0336	3
$\:{FR}_{4}$($\:{DR}_{4}$)	0.3416	0.0351	2
$\:{FR}_{5}$($\:{DR}_{5}$)	0.4444	0.0322	4
$\:{FR}_{6}$($\:{DR}_{6}$)	0.2630	0.0166	5
$\:{FR}_{7}({DR}_{7})$	0.9140	0.0479	1
$\:{FR}_{8}$($\:{DR}_{8}$)	0.1432	0.0060	9
$\:{FR}_{9}({DR}_{9})$	0.1127	0.0102	8

## Data validation and discussion

### Data validation

In this study, we conducted user feedback surveys to validate the DR against user requirements and used expert scoring and data analysis to verify the rationality of the FDR and FSR. For this, multiple domain experts were invited to evaluate each design requirement and obtain their corresponding FDR. Subsequently, the FSR was calculated by analyzing the existing technical and resource capabilities of the company. We primarily focused on the overlapping regions between FDR and FSR, as the overlap directly reflects the degree of alignment between the design and requirements. Theoretically, a larger overlapping region indicates a higher match between design needs and the company’s technological capabilities, thus increasing the likelihood of meeting user expectations. However, the overlapping region served as a single factor in the final ranking of the design requirements. Other metrics, such as IC, were incorporated to comprehensively evaluate priorities. For example, while the modular design ($\:{FR}_{5}$) exhibited a large overlapping area, the lightweight structure ($\:{FR}_{3}$) ranked higher in the final results. This discrepancy can be attributed to several factors:

(1) Impact of IC: The overlapping region reflects only the alignment between the design requirements and the company’s existing capabilities. In contrast, the IC value considers the complexities and uncertainties in satisfying the requirement. As the IC value reflects the overall alignment of the design with the user needs, it was given greater weight in the final rankings, causing it to influence the priority order of certain requirements.

(2) Complexity of design requirements: Some design requirements (e.g., material processes) may not rank high in the overlapping area but are complex and can significantly impact user satisfaction. Consequently, these requirements were assigned higher weights during comprehensive ranking, resulting in a better final performance.

(3) Multi-dimensional considerations: The final rankings did not rely on a single metric, such as the overlapping region, but also incorporated factors like user experience, technical feasibility, and resource allocation. Therefore, while some design requirements ranked high based on the overlapping region, other considerations (e.g., cost or ease of operation) influenced their relative position in the final rankings.

By employing this comprehensive evaluation approach, our results not only emphasize the alignment of the design requirements with the company’s capabilities but also balance user needs and technical feasibility, thus yielding a prioritized list of optimal design solutions.

### Discussion

This study analyzed the user requirements for SHAs, identifying several critical improvement metrics and proposing design optimization directions based on the Kano-QFD model. Our analysis highlighted 9 key factors influencing user satisfaction and quantified the alignment between user expectations and the company’s design capabilities by evaluating the overlapping areas between the FDR and FSR. The IC values were calculated and weighted according to the QFD-derived importance scores, resulting in a final prioritization of design needs. This analysis clarified the priorities among design requirements in terms of user satisfaction and provided actionable improvement directions.

(1) Environmental mode audio noise reduction ($\:{FR}_{7}$): The weighted score for environmental mode audio noise reduction ranked the highest (0.0479). Its high IC value (0.9140) indicates a significant gap between the current design and the user’s actual experience, reflecting an urgent unmet need for noise reduction among elderly users in complex environments. Suggested optimization direction: Enhance multi-scenario noise recognition algorithms and develop adaptive environmental sound field adjustment technologies to improve speech clarity in outdoor or noisy environments.

(2) High-capacity battery and battery management system ($\:{FR}_{4}$): Long battery life ranked second with a weighted score of 0.0351, indicating that users highly value prolonged SHA use. The IC value (0.3416) reveals a considerable gap between current design and user expectations, indicating room for further improvement. Future enhancements could optimize the battery management system to extend battery life without increasing SHA weight, thus addressing user needs for prolonged use and improving overall user satisfaction. In the future, alternative energy solutions such as wireless charging and solar power can be explored to extend the battery life of hearing aids.

(3) Lightweight structure ($\:{FR}_{3}$): This factor ranked the third, with a weighted score of 0.0336. Its IC value of 0.3219 indicated a significant mismatch between the design requirement and user expectations. Therefore, lightweight SHA design is at the top of the optimization priority, as it reduces wearing fatigue, especially for elderly users who wear it for prolonged periods. Finite Element Analysis (FEA) should be employed to evaluate the trade-offs among weight, strength, and cost for different materials such as titanium alloys and carbon fiber.

(4) Modular design ($\:{FR}_{5}$): This factor had a weighted score of 0.0322 and an IC value of 0.4444, again indicating a notable gap between current designs and user expectations. Modular design enhances ease of maintenance and customization; however, it must be in tandem with the requirements for lightweight structure and material processes to avoid increasing the overall product weight or material costs. Future improvements should aim to find a balance between cost control and modular functionality to better meet user needs.

(5) Audio components and acoustic design ($\:{FR}_{6}$): This factor had a weighted score of 0.0166, and a relatively high IC value of 0.2630, suggesting that the current design is closer to user expectations but can be improved further. Audio quality is critical for the hearing experience of elderly users. Enhancing sound clarity and reducing distortion and echo issues can bridge this gap and significantly improve overall user satisfaction. Thus, audio quality optimization is a key focus area for SHA improvement.

(6) Other factors ($\:{FR}_{2}$, durable high-strength plastics; $\:{FR}_{1}$, modern aesthetic design language; $\:{FR}_{9}$, easy-to-remove components; and $\:{FR}_{8}$, simple button interaction): Although these factors rank lower in the overall priority, addressing aspects such as simplifying operations, selecting appropriate materials, improving maintenance convenience, and maintaining aesthetic appeal will make the product more user-friendly and accessible for elderly users and enhance overall user satisfaction.

To reveal the prioritization mechanism of Functional Requirements (FRs), this study develops a quantitatively driven decision-making framework by integrating Information Content (IC value) with techno-economic contradiction analysis. As shown in Table 14, functional requirements are classified into three categories: “Core Innovation,” “Differentiated Competition,” and “Supportive Function.” This classification is based not only on the IC value but also on the solvability of technical challenges and the marginal benefits of corresponding engineering strategies. For example, although the environmental mode audio noise reduction (FR_7_) has the highest IC value (0.9140), its technical challenges mainly lie in noise recognition algorithms and environmental adaptability, thus requiring optimization between audio clarity and noise reduction effectiveness across various scenarios. In contrast, although high-strength plastic (FR_4_) has a slightly lower IC value (0.4316), its improvement potential is constrained by energy density limitations and the complexity of optimizing battery management system (BMS) algorithms. This makes it especially important to balance battery life and intelligent system control. This classification system provides a transparent standard for resource allocation: core innovation functions must be supported by mature technologies, while supportive functions can be maintained with minimal investment to ensure basic user experience. By integrating quantitative metrics with engineering evidence, the proposed framework eliminates the subjectivity inherent in traditional prioritization methods and addresses the enduring trade-off between “ideal performance” and “feasible implementation” in complex system design.

**Table 14:** Technical evaluation and prioritization matrix of functional Requirements.

Functional requirement	Rank	Technical Challenge	Engineering Strategy	Priority category
$\:{FR}_{7}$	1	Heavy hardware dependence; high frequency of use	Lightweight software combined with hardware reuse	Core Innovation
$\:{FR}_{4}$	2	Bottlenecks in energy density; difficulty in optimizing BMS algorithms	AI-based predictive maintenance; preliminary research into solid-state batteries; explore alternative energy solutions	Core Innovation
$\:{FR}_{3}$	3	Conflict between material strength and cost; high process complexity	Use Finite Element Analysis (FEA) to evaluate trade-offs among weight, strength, and cost for materials such as titanium alloys and carbon fiber.	Core Innovation
$\:{FR}_{5}$	4	Decreased interface strength; sacrifice of compactness	Standardized interfaces; partial modularization	Differentiated Competition
$\:{FR}_{6}$	5	High-cost components offer low marginal benefits	Directional sound field technology; selective investment	Differentiated Competition
$\:{FR}_{2}$	6	High cost (e.g., PEEK), limitations in recycling technologies	Glass fiber reinforced plastic (GFRP), cost reduction via modification technologies	Supportive Function
$\:{FR}_{1}$	7	Conflict between aesthetics and functionality; cost-sensitive	Conform to structural design; adopt minimalist approach	Supportive Function
$\:{FR}_{9}$	8	Risk of structural weakening; limited applicability across scenarios	Quick-release design for key components (e.g., battery compartment)	Supportive Function
$\:{FR}_{8}$	9	Declining demand under the trend of touchscreens	Conform to structural design; adopt minimalist approach	Supportive Function

Compared to traditional QFD methods, this study used a combination of the Kano-QFD + FAD method to analyze vague and uncertain user requirements. Traditional methods perform well with quantitative standards but show limitations when addressing products like SHAs, which involve various subjective perceptions. Fuzzy design provides greater flexibility in handling the diverse needs of elderly users, especially when the requirements are not clearly articulated in natural language.

This study has a few limitations, primarily in the scope of data collection. The data was obtained solely through online questionnaires, potentially overlooking the needs of SHA users who are less familiar with the internet or online surveys. This could introduce biases in the findings, making them less representative of the entire SHA user population. Future studies should expand the sample size and adopt offline collection methods, such as face-to-face interviews and telephone surveys, to increase sample diversity. This would enhance the representativeness of the data, making it applicable to a broader range of senior users while incorporating more real-world user feedback to validate the applicability of the framework. In addition, further exploration is required to effectively implement FAD in practical design processes. Considering the challenges that senior users face in expressing fuzzy needs, future efforts could involve standardization of FAD implementation steps through case studies, ensuring more accurate translation of user needs into specific parameters. Moreover, integrating artificial intelligence technologies could automate the processing of user requirement data. Combining natural language processing and machine learning algorithms could facilitate automatic analysis of user feedback and demand classification, thus reducing manual operations while improving analytical efficiency and accuracy.

Future applications of this framework could extend to diverse products, such as smart health watches and remote medical devices, allowing the development of multi-domain design strategies. Furthermore, product design in other industries may involve more diverse user needs and complex technical requirements, necessitating further exploration of the framework’s adaptability in demand classification and priority determination to ensure its feasibility in other contexts. Subsequent research could determine the effectiveness of the framework across different product categories and investigate the integration of Kano-QFD and FAD to suit various product types, thereby enhancing the framework’s generalizability.

## Conclusion

In this study, we developed a two-stage framework combining the Kano-QFD model and FAD, establishing an adaptive user satisfaction evaluation tool for designing SHAs. The framework effectively identifies and translates the ambiguous needs of elderly users to obtain a precise product design. Additionally, design range were optimized via validation through expert scoring and data analysis. The practicality and efficacy of this framework were verified, especially in resource-constrained scenarios, helping businesses optimize product development processes and enhancing the elderly user experience.

Using the top-ranked requirement as an example, the Kano model categorized user requirements and calculated a “better” coefficient of 49.69% and a “worse” coefficient of − 27.04% for the adaptability (C34) sub-criteria. Through QFD analysis, C42 was translated into the automatic adjustment function and personalized customization (H10) engineering element, with an initial QFD weight of 5.24%. Using AD theory, H10 was further translated into FRs and DR. The FDR and FSR areas for $\:{FR}_{7}$ calculated using the Kano model and expert scoring reveal an overlap area of 0.0663, indicating a high level of alignment between design and user requirements. Finally, IC value analysis reveals the highest IC value of 0.9140 for $\:{FR}_{7}$. By optimizing these key requirements, we validated the rationality of the proposed framework and significantly improved user satisfaction.

Overall, the two-stage user satisfaction framework based on the Kano-QFD model and FAD not only addresses user satisfaction but also fosters user loyalty. This framework lays a theoretical foundation for the development of smart products tailored to the elderly and demonstrates broad application potential. As demand for smart wearable devices among elderly users continues to grow, the proposed integrated approach can be extended beyond SHAs to other senior-assistive products, such as health monitoring devices and smart home technologies. With continuous refinement and wider adoption, this framework holds the potential to offer valuable insights across a range of industries.

## Supplementary Information

Below is the link to the electronic supplementary material.

Supplementary Material 1

Supplementary Material 2

## Data Availability

Data is provided within the manuscript or supplementary information files.
