# Supplementary material for: Optimization strategies for elderly hearing aid user satisfaction based on the Kano QFD integrated model and FAD theory
Source: Sci Rep. 2025 Oct 2;15:34441. doi: 10.1038/s41598-025-17546-5 (PMC12491538; doi:10.1038/s41598-025-17546-5)
Supplement: Supplementary file 1 — Supplementary Material 1 [file 41598_2025_17546_MOESM1_ESM.docx]

Kano Model-Based Functional Survey Questionnaire for Elderly Hearing Aid Headphones

1. Your Gender [Single Choice Question]

| Options | Subtotal | proportion |
| --- | --- | --- |
| male | 179 | 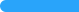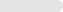55.76% |
| female | 142 | 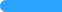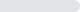44.24% |
| Valid entries | 321 |  |

2. Have you ever used hearing-related products (including hearing aids)? [Single Choice Question]

| Options | Subtotal | proportion |
| --- | --- | --- |
| Yes | 210 | 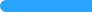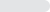65.42% |
| No | 111 | 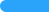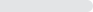34.58% |
| Valid entries | 321 |  |

3. Your age [Single Choice Question]

| Options | Subtotal | proportion |
| --- | --- | --- |
| 60-69 | 83 | 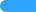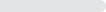25.86% |
| 70-79 | 108 | 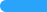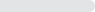33.64% |
| 80-89 | 86 | 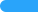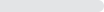26.79% |
| 90 and above | 44 | 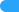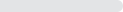13.71% |
| Valid entries | 321 |  |

4. Does the elderly hearing aid have a lightweight design? [Single Choice Question]

The average score for this matrix question: 2.88

| Question \ Options | Very attractive | Must be | Indifferent | Acceptable | Not attractive | Average score |
| --- | --- | --- | --- | --- | --- | --- |
| If this feature is present, how would you rate it? | 82(25.55%) | 150(46.73%) | 80(24.92%) | 6(1.87%) | 3(0.93%) | 3.94 |
| If this feature is absent, how would you rate it? | 12(3.74%) | 3(0.93%) | 73(22.74%) | 62(19.31%) | 171(53.27%) | 1.83 |
| Subtotal | 94(14.64%) | 153(23.83%) | 153(23.83%) | 68(10.59%) | 174(27.1%) | 2.88 |

5. Does the elderly hearing aid have good tactile feedback? [Single Choice Question]

The average score for this matrix question:**3.05**

| Question \ Options | Very attractive | Must be | Indifferent | Acceptable | Not attractive | Average score |
| --- | --- | --- | --- | --- | --- | --- |
| If this feature is present, how would you rate it? | 163(50.78%) | 81(25.23%) | 66(20.56%) | 7(2.18%) | 4(1.25%) | 4.22 |
| If this feature is absent, how would you rate it? | 12(3.74%) | 4(1.25%) | 71(22.12%) | 80(24.92%) | 154(47.98%) | 1.88 |
| Subtotal | 175(27.26%) | 85(13.24%) | 137(21.34%) | 87(13.55%) | 158(24.61%) | 3.05 |

6. Does the elderly hearing aid have a compact size? [Single Choice Question]

The average score for this matrix question:**3.18**

| Question \ Options | Very attractive | Must be | Indifferent | Acceptable | Not attractive | Average score |
| --- | --- | --- | --- | --- | --- | --- |
| If this feature is present, how would you rate it? | 155(48.29%) | 94(29.28%) | 69(21.5%) | 0(0%) | 3(0.93%) | 4.24 |
| If this feature is absent, how would you rate it? | 3(0.93%) | 1(0.31%) | 117(36.45%) | 111(34.58%) | 89(27.73%) | 2.12 |
| Subtotal | 158(24.61%) | 95(14.8%) | 186(28.97%) | 111(17.29%) | 92(14.33%) | 3.18 |

7. Does the elderly hearing aid have soft and comfortable ear tips? [Single Choice Question]

The average score for this matrix question:**2.92**

| Question \ Options | Very attractive | Must be | Indifferent | Acceptable | Not attractive | Average score |
| --- | --- | --- | --- | --- | --- | --- |
| If this feature is present, how would you rate it? | 102(31.78%) | 133(41.43%) | 82(25.55%) | 0(0%) | 4(1.25%) | 4.02 |
| If this feature is absent, how would you rate it? | 4(1.25%) | 0(0%) | 92(28.66%) | 63(19.63%) | 162(50.47%) | 1.82 |
| Subtotal | 106(16.51%) | 133(20.72%) | 174(27.1%) | 63(9.81%) | 166(25.86%) | 2.92 |

8. Does the elderly hearing aid have a preferred color design? [Single Choice Question]

The average score for this matrix question:**3.05**

| Question \ Options | Very attractive | Must be | Indifferent | Acceptable | Not attractive | Average score |
| --- | --- | --- | --- | --- | --- | --- |
| If this feature is present, how would you rate it? | 183(57.01%) | 72(22.43%) | 63(19.63%) | 0(0%) | 3(0.93%) | 4.35 |
| If this feature is absent, how would you rate it? | 3(0.93%) | 0(0%) | 74(23.05%) | 82(25.55%) | 162(50.47%) | 1.75 |
| Subtotal | 186(28.97%) | 72(11.21%) | 137(21.34%) | 82(12.77%) | 165(25.7%) | 3.05 |

9. Does the elderly hearing aid have a preferred design style? [Single Choice Question]

The average score for this matrix question:**3.21**

| Question \ Options | Very attractive | Must be | Indifferent | Acceptable | Not attractive | Average score |
| --- | --- | --- | --- | --- | --- | --- |
| If this feature is present, how would you rate it? | 163(50.78%) | 88(27.41%) | 66(20.56%) | 0(0%) | 4(1.25%) | 4.26 |
| If this feature is absent, how would you rate it? | 4(1.25%) | 1(0.31%) | 123(38.32%) | 107(33.33%) | 86(26.79%) | 2.16 |
| Subtotal | 167(26.01%) | 89(13.86%) | 189(29.44%) | 107(16.67%) | 90(14.02%) | 3.21 |

10. Does the elderly hearing aid have a design that reduces the stigma of use (so that others can't tell you are using a hearing aid)? [Single Choice Question]

The average score for this matrix question:**3**

| Question \ Options | Very attractive | Must be | Indifferent | Acceptable | Not attractive | Average score |
| --- | --- | --- | --- | --- | --- | --- |
| If this feature is present, how would you rate it? | 85(26.48%) | 84(26.17%) | 139(43.3%) | 1(0.31%) | 12(3.74%) | 3.71 |
| If this feature is absent, how would you rate it? | 12(3.74%) | 0(0%) | 139(43.3%) | 88(27.41%) | 82(25.55%) | 2.29 |
| Subtotal | 97(15.11%) | 84(13.08%) | 278(43.3%) | 89(13.86%) | 94(14.64%) | 3 |

11. Does the elderly hearing aid have a simple and reasonable button operation? [Single Choice Question]

The average score for this matrix question:**3.07**

| Question \ Options | Very attractive | Must be | Indifferent | Acceptable | Not attractive | Average score |
| --- | --- | --- | --- | --- | --- | --- |
| If this feature is present, how would you rate it? | 172(53.58%) | 67(20.87%) | 73(22.74%) | 5(1.56%) | 4(1.25%) | 4.24 |
| If this feature is absent, how would you rate it? | 8(2.49%) | 8(2.49%) | 69(21.5%) | 93(28.97%) | 143(44.55%) | 1.89 |
| Subtotal | 180(28.04%) | 75(11.68%) | 142(22.12%) | 98(15.26%) | 147(22.9%) | 3.07 |

12. Does the elderly hearing aid have excellent sound quality? [Single Choice Question]

The average score for this matrix question:**2.93**

| Question \ Options | Very attractive | Must be | Indifferent | Acceptable | Not attractive | Average score |
| --- | --- | --- | --- | --- | --- | --- |
| If this feature is present, how would you rate it? | 95(29.6%) | 122(38.01%) | 92(28.66%) | 9(2.8%) | 3(0.93%) | 3.93 |
| If this feature is absent, how would you rate it? | 8(2.49%) | 7(2.18%) | 91(28.35%) | 66(20.56%) | 149(46.42%) | 1.94 |
| Subtotal | 103(16.04%) | 129(20.09%) | 183(28.5%) | 75(11.68%) | 152(23.68%) | 2.93 |

13. Does the elderly hearing aid have long-lasting battery life? [Single Choice Question]

The average score for this matrix question:**3.21**

| Question \ Options | Very attractive | Must be | Indifferent | Acceptable | Not attractive | Average score |
| --- | --- | --- | --- | --- | --- | --- |
| If this feature is present, how would you rate it? | 159(49.53%) | 97(30.22%) | 60(18.69%) | 1(0.31%) | 4(1.25%) | 4.26 |
| If this feature is absent, how would you rate it? | 4(1.25%) | 0(0%) | 116(36.14%) | 120(37.38%) | 81(25.23%) | 2.15 |
| Subtotal | 163(25.39%) | 97(15.11%) | 176(27.41%) | 121(18.85%) | 85(13.24%) | 3.21 |

14. Does the elderly hearing aid adapt to different environments during use? [Single Choice Question]

The average score for this matrix question:**3.18**

| Question \ Options | Very attractive | Must be | Indifferent | Acceptable | Not attractive | Average score |
| --- | --- | --- | --- | --- | --- | --- |
| If this feature is present, how would you rate it? | 158(49.22%) | 88(27.41%) | 71(22.12%) | 1(0.31%) | 3(0.93%) | 4.24 |
| If this feature is absent, how would you rate it? | 3(0.93%) | 0(0%) | 119(37.07%) | 113(35.2%) | 86(26.79%) | 2.13 |
| Subtotal | 161(25.08%) | 88(13.71%) | 190(29.6%) | 114(17.76%) | 89(13.86%) | 3.18 |

15. Does the elderly hearing aid undergo strict testing and verification during the production process? [Single Choice Question]

The average score for this matrix question:**3.02**

| Question \ Options | Very attractive | Must be | Indifferent | Acceptable | Not attractive | Average score |
| --- | --- | --- | --- | --- | --- | --- |
| If this feature is present, how would you rate it? | 105(32.71%) | 86(26.79%) | 124(38.63%) | 2(0.62%) | 4(1.25%) | 3.89 |
| If this feature is absent, how would you rate it? | 4(1.25%) | 0(0%) | 128(39.88%) | 94(29.28%) | 95(29.6%) | 2.14 |
| Subtotal | 109(16.98%) | 86(13.4%) | 252(39.25%) | 96(14.95%) | 99(15.42%) | 3.02 |

16. Does the elderly hearing aid have a high durability and high-performance material manufacturing process? [Single Choice Question]

The average score for this matrix question:**3.01**

| Question \ Options | Very attractive | Must be | Indifferent | Acceptable | Not attractive | Average score |
| --- | --- | --- | --- | --- | --- | --- |
| If this feature is present, how would you rate it? | 102(31.78%) | 96(29.91%) | 118(36.76%) | 2(0.62%) | 3(0.93%) | 3.91 |
| If this feature is absent, how would you rate it? | 3(0.93%) | 0(0%) | 129(40.19%) | 89(27.73%) | 100(31.15%) | 2.12 |
| Subtotal | 105(16.36%) | 96(14.95%) | 247(38.47%) | 91(14.17%) | 103(16.04%) | 3.01 |

17. Does the elderly hearing aid have a sturdy internal structure? [Single Choice Question]

The average score for this matrix question:**3.18**

| Question \ Options | Very attractive | Must be | Indifferent | Acceptable | Not attractive | Average score |
| --- | --- | --- | --- | --- | --- | --- |
| If this feature is present, how would you rate it? | 154(47.98%) | 83(25.86%) | 80(24.92%) | 0(0%) | 4(1.25%) | 4.19 |
| If this feature is absent, how would you rate it? | 4(1.25%) | 0(0%) | 126(39.25%) | 108(33.64%) | 83(25.86%) | 2.17 |
| Subtotal | 158(24.61%) | 83(12.93%) | 206(32.09%) | 108(16.82%) | 87(13.55%) | 3.18 |

18. Does the elderly hearing aid have a price that is acceptable to consumers? [Single Choice Question]

The average score for this matrix question:**3.22**

| Question \ Options | Very attractive | Must be | Indifferent | Acceptable | Not attractive | Average score |
| --- | --- | --- | --- | --- | --- | --- |
| If this feature is present, how would you rate it? | 160(49.84%) | 75(23.36%) | 82(25.55%) | 0(0%) | 4(1.25%) | 4.21 |
| If this feature is absent, how would you rate it? | 4(1.25%) | 1(0.31%) | 122(38.01%) | 131(40.81%) | 63(19.63%) | 2.23 |
| Subtotal | 164(25.55%) | 76(11.84%) | 204(31.78%) | 131(20.4%) | 67(10.44%) | 3.22 |

19. Does the elderly hearing aid have a low manufacturing cost? [Single Choice Question]

The average score for this matrix question:**3.02**

| Question \ Options | Very attractive | Must be | Indifferent | Acceptable | Not attractive | Average score |
| --- | --- | --- | --- | --- | --- | --- |
| If this feature is present, how would you rate it? | 92(28.66%) | 88(27.41%) | 132(41.12%) | 5(1.56%) | 4(1.25%) | 3.81 |
| If this feature is absent, how would you rate it? | 4(1.25%) | 0(0%) | 142(44.24%) | 93(28.97%) | 82(25.55%) | 2.22 |
| Subtotal | 96(14.95%) | 88(13.71%) | 274(42.68%) | 98(15.26%) | 86(13.4%) | 3.02 |

20. Does the elderly hearing aid have a low maintenance cost? [Single Choice Question]

The average score for this matrix question:**3.05**

| Question \ Options | Very attractive | Must be | Indifferent | Acceptable | Not attractive | Average score |
| --- | --- | --- | --- | --- | --- | --- |
| If this feature is present, how would you rate it? | 165(51.4%) | 75(23.36%) | 73(22.74%) | 5(1.56%) | 3(0.93%) | 4.23 |
| If this feature is absent, how would you rate it? | 7(2.18%) | 8(2.49%) | 82(25.55%) | 61(19%) | 163(50.78%) | 1.86 |
| Subtotal | 172(26.79%) | 83(12.93%) | 155(24.14%) | 66(10.28%) | 166(25.86%) | 3.05 |

The sum of the average scores for the questions: 104.35.

This study involving human participants was conducted in compliance with ethical guidelines and regulations. The research has been approved by the ethics committee.

All methods were performed in accordance with relevant ethical standards and regulations, including the principles outlined in the Declaration of Helsinki. Informed consent was obtained from all participants prior to their involvement in the study. Participants were provided with detailed information about the study's purpose, procedures, and their rights, ensuring voluntary participation without any coercion.

Hongik university
