# Supplementary material for: Optimization strategies for elderly hearing aid user satisfaction based on the Kano QFD integrated model and FAD theory
Source: Sci Rep. 2025 Oct 2;15:34441. doi: 10.1038/s41598-025-17546-5 (PMC12491538; doi:10.1038/s41598-025-17546-5)
Supplement: Supplementary file 2 — Supplementary Material 2 [file 41598_2025_17546_MOESM2_ESM.docx]

**SPSS Analysis Report**

Validity Analysis -1

| Item | Factor 1 | Factor 2 | Factor 3 | Factor 4 | Factor 5 | Factor 6 | Factor 7 | Communality |
| --- | --- | --- | --- | --- | --- | --- | --- | --- |
| If this feature is present, how would you rate it? | -0.04 | -0.19 | -0.08 | -0.31 | 0.64 | -0.00 | 0.04 | 0.556 |
| If this feature is absent, how would you rate it? | -0.20 | 0.73 | -0.03 | 0.16 | -0.11 | 0.02 | 0.05 | 0.610 |
| If this feature is present, how would you rate it? | 0.37 | -0.62 | -0.03 | 0.10 | 0.26 | -0.03 | 0.11 | 0.619 |
| If this feature is absent, how would you rate it? | -0.11 | 0.79 | 0.14 | 0.12 | 0.02 | 0.05 | -0.15 | 0.691 |
| If this feature is present, how would you rate it? | 0.69 | -0.19 | 0.06 | -0.11 | -0.08 | -0.12 | 0.09 | 0.552 |
| If this feature is absent, how would you rate it? | -0.07 | 0.07 | 0.06 | 0.62 | -0.36 | 0.04 | 0.18 | 0.567 |
| If this feature is present, how would you rate it? | 0.10 | -0.13 | -0.16 | -0.34 | 0.48 | 0.17 | 0.53 | 0.702 |
| If this feature is absent, how would you rate it? | -0.74 | 0.17 | 0.00 | 0.14 | 0.01 | -0.02 | 0.18 | 0.627 |
| If this feature is present, how would you rate it? | 0.62 | -0.10 | -0.05 | 0.16 | 0.24 | -0.19 | 0.23 | 0.577 |
| If this feature is absent, how would you rate it? | -0.40 | 0.17 | 0.06 | 0.11 | 0.23 | 0.66 | -0.04 | 0.687 |
| If this feature is present, how would you rate it? | 0.72 | -0.19 | -0.03 | -0.12 | 0.02 | 0.09 | -0.00 | 0.579 |
| If this feature is absent, how would you rate it? | -0.17 | -0.13 | 0.14 | 0.69 | 0.09 | 0.17 | -0.31 | 0.672 |
| If this feature is present, how would you rate it? | -0.14 | -0.06 | -0.44 | -0.06 | 0.26 | -0.58 | -0.02 | 0.614 |
| If this feature is absent, how would you rate it? | 0.17 | 0.04 | 0.42 | 0.21 | -0.16 | 0.53 | 0.11 | 0.578 |
| If this feature is present, how would you rate it? | 0.31 | -0.64 | -0.04 | -0.02 | 0.17 | 0.01 | 0.31 | 0.631 |
| If this feature is absent, how would you rate it? | -0.15 | 0.74 | 0.06 | 0.01 | -0.13 | 0.13 | -0.06 | 0.609 |
| If this feature is present, how would you rate it? | 0.04 | -0.20 | -0.07 | -0.21 | 0.75 | -0.07 | 0.04 | 0.655 |
| If this feature is absent, how would you rate it? | -0.24 | 0.74 | -0.02 | 0.23 | 0.04 | 0.04 | 0.10 | 0.679 |
| If this feature is present, how would you rate it? | 0.64 | -0.33 | -0.13 | 0.08 | 0.03 | -0.07 | 0.24 | 0.607 |
| If this feature is absent, how would you rate it? | -0.13 | 0.14 | 0.15 | 0.69 | -0.05 | -0.09 | 0.01 | 0.539 |
| If this feature is present, how would you rate it? | 0.76 | -0.15 | 0.02 | -0.03 | 0.04 | -0.02 | -0.04 | 0.599 |
| If this feature is absent, how would you rate it? | -0.02 | 0.18 | 0.06 | 0.62 | -0.30 | 0.01 | 0.06 | 0.516 |
| If this feature is present, how would you rate it? | 0.12 | 0.12 | -0.71 | -0.05 | 0.01 | -0.03 | 0.14 | 0.557 |
| If this feature is absent, how would you rate it? | 0.00 | 0.09 | 0.72 | 0.13 | 0.06 | -0.01 | -0.07 | 0.556 |
| If this feature is present, how would you rate it? | 0.10 | 0.21 | -0.65 | -0.04 | 0.12 | -0.16 | 0.14 | 0.542 |
| If this feature is absent, how would you rate it? | 0.02 | 0.04 | 0.69 | 0.10 | -0.12 | 0.07 | 0.37 | 0.639 |
| If this feature is present, how would you rate it? | 0.70 | -0.22 | -0.07 | -0.18 | -0.14 | 0.12 | 0.03 | 0.618 |
| If this feature is absent, how would you rate it? | 0.04 | 0.20 | 0.17 | 0.66 | -0.02 | 0.10 | -0.25 | 0.579 |
| If this feature is present, how would you rate it? | 0.74 | -0.14 | 0.07 | -0.04 | 0.07 | 0.02 | -0.05 | 0.580 |
| If this feature is absent, how would you rate it? | -0.10 | 0.09 | 0.03 | 0.55 | -0.31 | 0.19 | 0.18 | 0.496 |
| If this feature is present, how would you rate it? | -0.11 | -0.16 | -0.62 | -0.14 | 0.05 | -0.19 | 0.19 | 0.512 |
| If this feature is absent, how would you rate it? | -0.00 | 0.17 | 0.74 | 0.06 | -0.01 | -0.02 | 0.17 | 0.605 |
| If this feature is present, how would you rate it? | 0.40 | -0.59 | 0.08 | -0.02 | 0.26 | -0.02 | -0.15 | 0.605 |
| If this feature is absent, how would you rate it? | -0.23 | 0.68 | 0.02 | 0.25 | -0.05 | -0.07 | 0.24 | 0.645 |
| Eigenvalue (before rotation) | 8.01 | 4.33 | 2.55 | 2.05 | 1.33 | 1.08 | 1.03 | - |
| Variance Explained % (Before Rotation) | 23.56% | 12.75% | 7.51% | 6.04% | 3.92% | 3.18% | 3.04% | - |
| Cumulative Variance Explained (%) (Before Rotation) | 23.56% | 36.31% | 43.81% | 49.85% | 53.77% | 56.95% | 59.99% | - |
| Eigenvalues (after rotation) | 4.85 | 4.53 | 3.40 | 3.11 | 2.00 | 1.34 | 1.16 | - |
| **Variance Explained % (After Rotation)** | 14.27% | 13.33% | 10.01% | 9.16% | 5.87% | 3.95% | 3.41% | - |
| **Cumulative Variance Explained % (after Rotation)** | 14.27% | 27.60% | 37.60% | 46.77% | 52.64% | 56.59% | 59.99% | - |
| Kaiser-Meyer-Olkin (KMO) Measure of Sampling Adequacy | 0.864 | | | | | | | - |
| Bartlett's Test of Sphericity | 4708.680 | | | | | | | - |
| degrees of freedom | 561.000 | | | | | | | - |
| *p*-value | - | | | | | | | - |

Validity Analysis -1

This study validated the questionnaire's effectiveness through factor analysis, extracting seven factors. The analysis revealed a Kaiser-Meyer-Olkin (KMO) measure of **0.864**, confirming the sample's suitability for factor analysis. Bartlett’s test of sphericity (**χ² = 4708.680**, df = 561, p < 0.001) further validated significant inter-variable correlations. Pre-rotation eigenvalues indicated that the first factor explained **23.56%** of the variance, followed by the second factor (**12.75%**), with cumulative variance reaching **59.99%**. Post-rotation results (e.g., Varimax) showed the first factor accounted for **14.27%** and the second for **13.33%**, maintaining the cumulative variance at **59.99%**, underscoring structural stability. Key items like " *If this feature is absent, how would you rate it?*" exhibited strong loadings on Factor 2 (**0.73**) and Factor 4 (**0.69**), while "*If this feature is present, how would you rate it?*" loaded prominently on Factor 5 (**0.75**). All items demonstrated communalities between **0.496–0.702**, reflecting robust alignment with latent factors. These results confirm the questionnaire’s validity for assessing target traits and provide a foundation for future research. Further validation of factor structure stability and reliability is recommended to enhance practical utility.

*In Kano model research, it is crucial to conduct separate reliability analyses for questionnaire data when features are present ("If present") versus absent ("If absent"). The two types of questions measure fundamentally distinct dimensions—"presence" reflects user satisfaction, while "absence" assesses tolerance—and merging these analyses would distort reliability metrics.* ***Therefore, we analyze these two categories separately to enhance the accuracy of reliability analysis****, ensuring that users' differentiated feedback on feature presence and absence can be independently validated. This approach provides a more robust data foundation for subsequent Kano classification and prioritization of requirements.*

Reliability Analysis-1

| Sample Size | Number of Items | Cronbach's Alpha coefficient |
| --- | --- | --- |
| 321 | 17 | 0.819 |

Reliability Analysis-1

In this study, we conducted reliability analysis on a questionnaire comprising 321 samples and 17 items. The results revealed a Cronbach's α coefficient of 0.819, indicating good internal consistency that exceeds the commonly accepted reliability threshold of 0.70. This suggests strong inter-item correlations for the measured construct. Specifically, a Cronbach's α of 0.819 demonstrates that the questionnaire items effectively capture the characteristics and attitudes of the research subjects. These findings imply that the questionnaire is well-designed and capable of reliably reflecting respondents' true perceptions. The high reliability also provides robust support for subsequent data analysis and the credibility of research outcomes.However, despite the favorable results, potential limitations such as sample representativeness and questionnaire applicability should be acknowledged. Future studies could expand sample size and diversity to further validate the questionnaire's reliability and validity.

Reliability Analysis -2

| Sample Size | Number of Items | Cronbach's Alpha coefficient |
| --- | --- | --- |
| 321 | 17 | 0.826 |

Reliability Analysis -2

In this study, we conducted a reliability analysis on a questionnaire with a sample size of 321, using 17 items to assess the internal consistency of the questionnaire. According to the analysis results, the Cronbach's α coefficient was **0.826**, indicating **good reliability**. The Cronbach's α coefficient typically ranges from 0 to 1, with higher values indicating stronger internal consistency of the questionnaire. Based on general standards, an α coefficient above **0.7** is considered acceptable, and above **0.8** is regarded as good. Therefore, the result of **0.826** in this study not only exceeds the acceptable threshold but also demonstrates the reliability of the questionnaire in measuring the target construct.

In conclusion, the questionnaire exhibits **high reliability** based on the sample size and number of items, providing a solid foundation for subsequent research. Future studies could further explore the **validity** of the questionnaire and its applicability across diverse populations.
